# Future trends and guidance for the triple bottom line and sustainability: a data driven bibliometric analysis

**DOI:** 10.1007/s11356-020-09284-0

**Published:** 2020-06-22

**Authors:** Ming-Lang Tseng, Chia-Hao Chang, Chun-Wei Remen Lin, Kuo-Jui Wu, Qing Chen, Li Xia, Bing Xue

**Affiliations:** 1 Institute of Innovation and Circular Economy, Asia University, Taichung, Taiwan; 2 Department of Medical Research, China Medical University Hospital, China Medical University, Taichung, Taiwan; 3 Faculty of Economic and Management, University Kebangsaan Malaysia, Bangi, Malaysia; 4 Department of Business Administration, Asia University, Taichung, Taiwan; 5 College of Management, Asia University, Taichung, Taiwan; 6 School of Management, National Taiwan University of Science and Technology, Taipei, 10607 Taiwan; 7 School of Business, Dalian University of Technology, Dalian, China; 8 School of Management, University of Science and Technology, Anhui, China; 9 Key Lab for Environmental Computation and Sustainability of Liaoning Province, Institute of Applied Ecology, Chinese Academy of Sciences, Shenyang, 110016 China

**Keywords:** Triple bottom line, Literature review, Bibliometric analysis, Sustainability, Sustainable development

## Abstract

This study conducts a comprehensive literature review of articles on the triple bottom line (TBL) published from January 1997 to September 2018 to provide significant insights and support to guide further discussion. There were three booms in TBL publications, occurring in 2003, 2011, and 2015, and many articles attempt to address the issue of sustainability by employing the TBL. This literature analysis includes 720, 132, and 58 articles from the Web of Science (WOS), Inspec, and Scopus databases, respectively, and reveals the gaps in existing research. To discover the barriers and points of overlap, these articles are categorized into six aspects of the TBL: economic, environmental, social, operations, technology, and engineering. Examining the top 3 journals in terms of published articles on each aspect reveals the research trends and gaps. The findings provide solid evidence confirming the argument that the TBL as currently defined is insufficient to cover the entire concept of sustainability. The social and engineering aspects still require more discussion to support the linkage of the TBL and to reinforce its theoretical basis. Additionally, to discover the gaps in the data sources, theories applied, methods adopted, and types of contributions, this article summarizes 82 highly cited articles covering each aspect. This article offers theoretical insights by identifying the top contributing countries, institutions, authors, keyword networks, and authorship networks to encourage scholars to push the current discussion further forward, and it provides practical insights to bridge the gap between theory and practice for enhancing the efficiency and effectiveness of improvements.

**Electronic supplementary material:**

The online version of this article (10.1007/s11356-020-09284-0) contains supplementary material, which is available to authorized users.

## Introduction

Elkington (1997) proposed the triple bottom line (TBL), comprising the planet, people, and profits, to address the issue of sustainability. The number of articles published on this subject increased slightly in 2003 after the Restriction of Hazardous Substances Directive and the Waste Electrical and Electronic Equipment Declaration were issued by the European Union. Academia and industry strived to identify ways to maintain economic growth while reducing negative impacts on the environment. However, despite the increasing number of articles, the components of the TBL were not sufficiently clear to address sustainability because the concept remained in a nascent stage (Bordass 2000; Jeurissen 2000). Subsequently, Carter and Rogers (2008) extended Elkington’s concept to economic, environmental, and social aspects and redefined the TBL as follows: “sustainability should hold economic performance, the natural environment and society at a broader level, and the intersection of social, environmental and economic activities can help organizations become engaged in activities that not only positively affect the natural environment and society but that also result in long-term economic benefits and competitive advantage for the firms.”

After Carter and Rogers (2008) redefined the TBL, the number of relevant articles boomed. Nevertheless, climate change accelerated, causing approximately 300,000 deaths and generating approximately USD 15.9 billion in losses worldwide in 2010. In that year, the European Union also suffered a serious debt crisis. These issues caused the number of published articles to decrease, although it also reflected the importance of the TBL balance. Carter and Easton (2011) attempted to address these issues and balance environmental, economic, and social performance by considering strategy, risk management, organizational culture, and transparency in their comprehensive evaluation. Freeman and Hasnaoui (2011) employed data on the UK, France, the USA, and Canada to explore a universal framework to address social issues in terms of corporate social responsibility. Moreover, the United Nations held a climate change conference in Durban, South Africa, to develop a new agreement to limit carbon emissions. After this conference, the number of articles on the TBL showed a steep growth. To promote the understanding of and precise actions toward sustainability development, the United Nations announced the 2030 agenda of sustainable development goals, which include no poverty, zero hunger, good health and well-being, quality education, gender equality, clean water and sanitation, affordable and clean energy, decent work, economic growth, strong industry, a climate of innovation, accessible infrastructure, reduced inequalities, sustainable cities and communities, responsible consumption and production, climate action, life below water, life on land, peace, justice, and linked institutions and partnerships to achieve these goals.

Although an increasing number of countries and areas are striving to identify an optimal solution to address these issues, arguments between academia and industry have emerged. For example, Wu et al. (2016) argued that economic, environmental, and social aspects do not sufficiently cover the entire concept of sustainability; thus, that study proposed four additional aspects (operations, resilience, long-term, and stakeholders) to promote further discussion. Tseng (2017) emphasized that sustainability issues are characterized by high complexity and uncertainty. To promote the quality and success of solutions, integrating qualitative information, quantitative data, and social media into the TBL discussion is required. Wu et al. (2018) found that the global electronics industry is attempting to develop sustainability and that the traditional aspects of the TBL are no longer sufficient to address sustainability issues, which must be addressed by the “overlapping bottom line (eco-efficiency, socioeconomic and socioenvironmental)” to obtain co-benefits.

Therefore, the objective of this article is to organize the major trends and gaps in the TBL discussion from the past two decades and to provide specific directions for future research to address sustainability. In addition, this article makes three contributions: (1) it summarizes the relevant theories to complete the TBL puzzle, (2) it explores possible approaches to enhancing the effectiveness and efficiency of applications, and (3) it specifies the gaps to promote future research. The remaining sections of this article are as follows. The data collection and cleaning procedures are presented in the “Theoretical background” section. The analytical results are classified into six major aspects, which are summarized in each subsection in the “Method” section. An in-depth discussion summarizing 82 articles that considers each aspect is presented in the “The insights from 82 highly cited articles” section. The “Conclusions” section provides the conclusions and research limitations.

## Theoretical background

Balancing the TBL toward sustainability involves an essentially dynamic and multidimensional concept (Alvarez et al. 2016). Carter and Rogers (2008) attempted to address sustainability issues by considering the economic, environmental, and social aspects; nevertheless, some articles have highlighted that these proposed aspects are insufficient to cover the entire concept of sustainability (Carter and Easton 2011; Wu et al. 2016; Wu et al. 2018). Although the operations aspect has been well discussed over the past two decades, the TBL still does not take it into account (Wu et al. 2016). However, sustainability performance can be improved effectively and efficiently through operational practices (Tahir and Darton 2010; Zailani et al. 2012). Some previous discussions on technology focused on the tradeoffs between sustainability and economic competitiveness (Kleindorfer et al. 2005). Porter (1999) argued that tough environmental standards can trigger innovation and the upgrading of technology and that properly constructed regulatory standards aimed at outcomes can also encourage companies to redesign their current technology. There are few articles related to engineering aspects; most of the existing articles focus on knowledge development and propose novel methods with descriptive and qualitative concentrations, while others analyze the possible optimization parameters to achieve continuous improvement in sustainability (Byggeth et al. 2007; Pusavec et al. 2010a, b; Xu and Liu 2013).

### Economic aspect

**Fig. 1:**
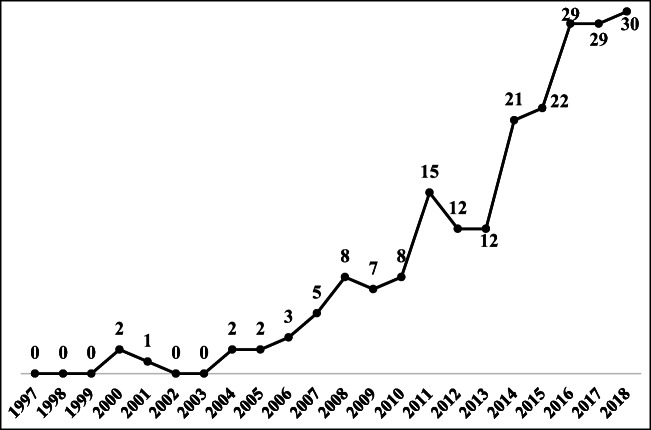
The trend in published TBL articles on the economic aspect

To better describe the evolutionary trajectory of the economic aspect of the TBL, the trend in published TBL articles on the economic aspect over the past two decades is shown in Fig. 5. Few TBL articles discussed the economic aspect to address sustainability during the initial stage, but a growth trend is observed, as increasingly more large businesses began to consider their sustainability initiatives in association with economic performance more seriously (Hashmi et al. 2014). Carter and Rogers (2008) demonstrated that firms that attempt to simultaneously maximize their performance across the three aspects of the TBL will outperform firms that attempt to achieve high levels of social and environmental performance without explicitly considering economic performance. Accordingly, Carter and Easton (2011) pointed out that rather than suggesting that firms identify and engage in social and environmental activities that will hopefully help, or at least not harm, economic performance, the TBL specifically guides managers to identify activities that improve economic performance and requires the avoidance of social and environmental activities that fall outside of this intersection. In the last 3 years, the number of TBL articles related to the economic aspect has shown more gradual growth, as the previous articles overemphasized economic benefits and neglected to address the economic practices that specifically balance the aspects of the TBL toward sustainability (Büyüközkan and Karabulut 2018; Bals and Tate 2017) (Fig. 1).

The most highly cited article, which was published by Carter and Rogers (2008), had 846 citations. In this article, the authors first introduced the concept of sustainability to the supply chain management field and demonstrated the relationships between environmental, social, and economic performance in the supply chain management context. The second most highly cited article was published by Carter and Easton (2011), and of the top 20 articles, it was the earliest article to address the economic aspect of the TBL by conducting a systematic literature review based on evaluated empirical sustainable supply chain management studies. However, the article by Norman and MacDonald (2004), which was the third most highly cited article, with 250 citations over the past two decades, argued that the TBL was an unhelpful addition to current discussions linking economic performance with corporate social responsibility.

Wu and Pagell (2011) developed a theory for balancing short-term profitability and long-term environmental sustainability while making decisions under uncertainty, and Hahn et al. (2015) proposed a systematic framework to analyze the tensions in corporate sustainability. However, most of the articles discussed the TBL by employing a qualitative method as the primary approach. Although Gleim et al. (2013) attempted to adopt a critical incident qualitative study and two quantitative studies to examine the factors of nongreen purchase behavior, the main data still came from a qualitative survey. Such data limitations might hinder the ability to solve economic issues when considering the TBL and might generate constraints in decision-making. Thus, several recent articles have strived to propose a hybrid method to integrate diverse data sources (including qualitative, quantitative, and social media data) to enhance the quality of the results and the accuracy of decision-making (Chan et al. 2016; Tseng 2017; Wu et al. 2017a, b).

### Environmental aspect

**Fig. 2:**
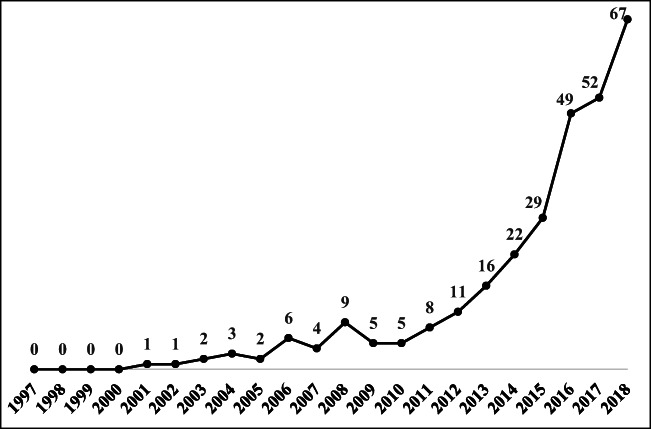
The trend in published TBL articles on the environmental aspect

Figure 2 presents the trend in published TBL articles on the environmental aspect, revealing slow growth during the 1997–2010 period. However, the number of publications since 2015 has boomed. In recent years, an increasing number of articles have emphasized the importance of the environment in the TBL discussion to achieve sustainability. Therefore, several studies designed systems to maximize environmental and social benefits rather than prioritizing economic growth, as these benefits might be a feature of a path to sustainability (Bocken et al. 2014; Jackson 2009). Moreover, Klewitz and Hansen (2014) explained that firms must make an effort to develop systematic management to balance environmental and social performance in a manner that is aligned with economic goals, offering a better understanding of TBL practices to firms as they pursue sustainability.

Although these articles have focused on expanding the sustainability concept, several articles have attempted to conduct a literature review to complete the definition and to explore future research. For example, Hahn and Kuehnen (2013) reviewed 178 articles from 1999 to 2011 related to business, management, and accounting; they found that the influence of variables related to environmental performance has been neglected and that the few existing studies considering this influence have provided inconsistent results. However, traditional small- and medium-sized enterprises have overemphasized environmental process innovations, which they considered to be the first step in conducting aggressive innovations. Thus, Klewitz and Hansen (2014) conducted a systematic review of sustainability-oriented innovative practices for small- and medium-sized enterprises to demonstrate the relationship between the environmental aspect and the TBL.

The majority of the 20 most highly cited articles attempted to structure a conceptual framework to guide sustainable development. For instance, Mori and Christodoulou (2012) emphasized the development of a new framework for the city sustainability index, yet this framework still needs to consider the total environmental impact instead of the environmental impact per capita, even though the environmental impact per capita is small due to the large population. Wiedmann et al. (2009) integrated all indicators related to environmental life cycle thinking (including carbon emissions, ecological and water footprinting) into a TBL accounting framework to construct a software package to help firms in the upstream supply chain to optimize their TBL, carbon footprint and services. Although these indicators have been well understood and selected as requirements in practice, addressing environmental issues to balance the TBL still involves an implicit decision-making process. Thus, the multicriteria decision-making (MCDM) approach is proposed to address these ambiguous sustainable decision-making problems, and it has proven to be an effective and efficient tool for guiding practices to pursue sustainability (Gulcin and Gizem 2012; Tseng 2009; Wu et al. 2016). Although this kind of article is missing from the top 20 list, the related discussion has grown increasingly in recent years.

### Social aspect

**Fig. 3:**
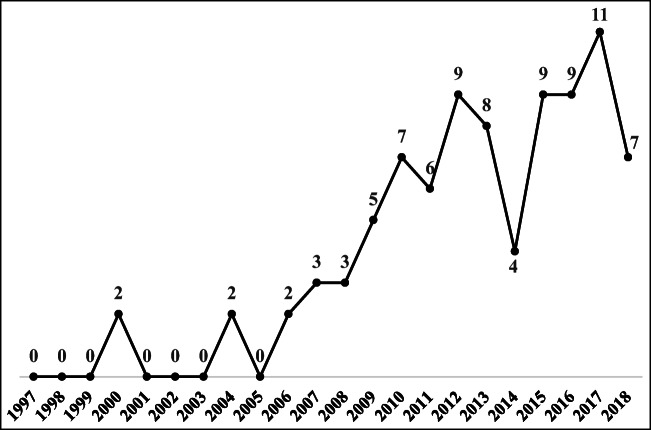
The trend in published TBL articles on the social aspect

As shown in Fig. 3, before 2006, very few TBL articles attempted to take the social aspect into account. However, since 2006, the number of published articles related to the social aspect has increased due to the emergent need to identify global social instability stemming from the European debt crisis, the threat of terrorism, the nuclear crisis, and other factors. Although the economic and environmental aspects of the TBL have been extensively covered by the proposed theories and practices based on previous efforts, the social aspect remains largely unknown (Tate and Bals 2018). Indeed, this study finds only 87 published articles discussing the social aspect, which is much lower than the number discussing the economic and environmental aspects, i.e., 208 and 292 articles, respectively. This result might be because the social aspect of the TBL plays only an intermediary role in the economic process and its effect on the environment (Svensson et al. 2018).

Norman and MacDonald (2004) emphasized that social performance or social impact can be measured objectively based on basic standard indicators; thus, firms are obligated to maximize the social bottom line. This article has the most citations: 250. Milne and Gray (2013), who were cited 164 times over the past 5 years, argued that the sustainability report of the Global Reporting Initiative lacks consideration of all stakeholders and cherry-picks elements of the news while overlooking the major social issues caused by a firm’s activities. Hahn et al. (2015) drew on the literature on strategic contradictions, tensions, and paradoxes to develop an integrated framework for identifying and characterizing the tensions in corporate sustainability aiming to address social considerations. These top 20 articles have strived to address the social aspect from a single view of the firm; however, although the government is partly responsible for solving social issues, discussion of the government was missing in these articles. Regarding the social aspect, holistic factors are still needed to reinforce the basis of cognizance and to reflect the real-world situation.

### Operations aspect

**Fig. 4:**
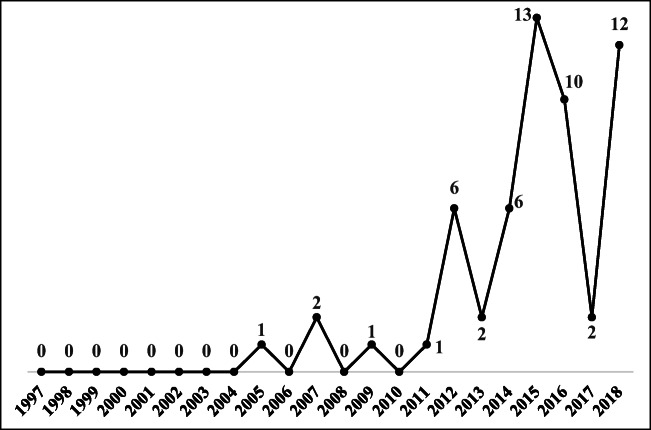
The trend in published TBL articles on the operations aspect

In dealing with sustainability issues, consideration of the operations aspect occurred later than consideration of the economic, environmental, and social aspects, as shown in Fig. 4. In the initial discussion of the TBL based on Carter and Rogers (2008), the operations aspect was not included in the TBL concept. Thus, almost no TBL articles took operations into account when addressing sustainability issues. Since 2011, the number of published TBL articles related to operations has increased, but it still presents a fluctuating trend. The classic article of Wu and Pagell (2011) discussed operations from the perspective of balancing short-term profitability and long-term environmental sustainability while making supply chain decisions under uncertainty. Wu et al. (2016) argued that covering the entire concept of sustainability based only on the TBL is insufficient; it is necessary to take operations into account to help firms achieve sustainability. Thus, the number of TBL articles related to the operations aspect has been increasing in recent years.

A total of 40% of highly cited articles were published in the *International Journal of Production Economics*. The most highly cited article is that by Kleindorfer et al. (2005), which was published in *Production and Operation Management* and cited 579 times over the past 13 years; it reviewed sustainability themes (such as lean operations, green operations, closed-loop supply chains and green product design) in the first 50 issues of *Production and Operation Management*. The conclusion was that both the theories and practices of operations management enable sustainability to be addressed. The next most highly cited article is that by Wu and Pagell (2011), published in the *Journal of Operation Management*; cited 210 times, it employs case studies that collected business models and operations along supply chains to identify how organizations incorporate environmental initiatives in supply chain management and operations decision-making. An article by Gimenez et al. (2012) is the third most highly cited article, with 174 citations. This article reviewed the literature on sustainable operations and tested the hypotheses to find that internal environmental initiatives have positive effects on achieving the TBL, while internal social initiatives only have positive effects on achieving social and environmental performance. Nevertheless, Wheeler et al. (2002) demonstrated that there is a large gap between top management and decision-making at the operational level when a firm is launching sustainable development. Thus, future TBL articles will need to explore the precise structure to guide the improvement of the operations aspect.

### Technology aspect

**Fig. 5:**
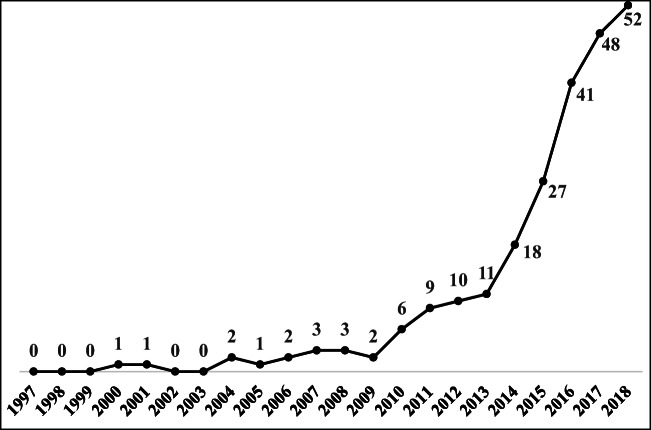
The trend in published TBL articles on the technology aspect

Very few articles have discussed the technology aspect in relation to the TBL, as shown in Fig. 5. Bordass (2000) is the first article to discuss the TBL in terms of technology, focusing on developing a system for assessing the costs and benefits of any building. Craig (2004) emphasized that technology is viewed as key to leading the changes necessary for a sustainable future. The published TBL articles related to technology show slow growth until 2015, which was a landmark year for artificial intelligence; the number of projects and software packages that were launched in this year reached 2700 items, and almost 71% of published articles invoking the technology aspect to address sustainability issues appeared after 2015. In other words, sustainability issues might be overcome through technological development.

In terms of the technology aspect, 60% of these highly cited articles are published in the *Journal of Cleaner Production*. These articles have 1348 citations, which means that each paper has an average of 11 annual citations over the past decade. Bocken et al. (2014) had the highest number of citations: 343. They discussed sustainable business model archetypes and grouped them into three types of business model innovations (technologically, socially, and organizationally oriented innovation). They found that technological innovation can effectively drive new business model innovations toward sustainability. With 223 citations, the second most highly cited paper was published by Govindan et al. (2013), who summarized a number of criteria, such as technology capability, resource consumption, and employment practices, to select sustainable suppliers by adopting the order of preference by similarity to ideal solution (TOPSIS) technique. Furthermore, Klewitz and Hansen (2014) are the third most frequently cited article, with 156 citations; they offered a specific illustration of the sustainability-oriented innovation practices of small- and medium-sized enterprises by conducting an extensive literature review of the 1987–2010 period and concluded that the technology factor plays an important role in the development of sustainability-oriented innovation. However, these articles made the connection only between the aspects of technology, the economy, and the environment. The linkage between technology and the social aspects is still missing in these discussions.

### Engineering aspect

**Fig. 6:**
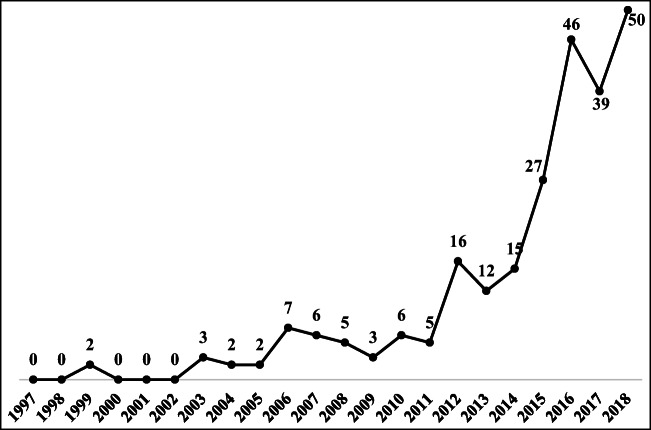
The trend in published TBL articles on the engineering aspect

As shown in Fig. 6, before 2003, almost no articles addressed the engineering aspect of the TBL. From 2003 to 2011, there appears to have been a gradual increase in the number of related articles. Oehlberg et al. (2010) designed a sustainable system for integrating the TBL concept into a freshman introductory engineering course at UC Berkeley. Álvarez et al. (2016) observed that engineering contains an increasing body of knowledge, motivated by the rising interest in process life cycle sustainability, which indicates that the TBL should take the engineering aspect into account in its comprehensive considerations to achieve sustainability. Thus, 46, 39, and 50 TBL articles related to the engineering aspect were published in 2016, 2017, and 2018, respectively; these articles account for almost 55% of the total number of related articles.

In the most highly cited article, with 579 citations, Kleindorfer et al. (2005) suggested reinforcing sustainable operations practices through design and life cycle analysis to strengthen the links between the economic, operations, and engineering aspects. Moreover, many creative approaches can contribute to developing sustainability by innovating the business model. Thus, in the second most highly cited article, with 343 citations, Bocken et al. (2014) identified the patterns and attributes of sustainable business model archetypes by conducting a comprehensive literature review in terms of business practices. A paper by Govindan et al. (2013) is the third most highly cited article, with 223 citations; this paper strives to explore sustainable supply chain initiatives and to demonstrate an effective model based on the TBL for selecting the optimal supplier by employing the fuzzy multicriteria approach.

Two articles that adopt reverse logistics to address the issue of the closed-loop supply chain require additional attention. Presley et al. (2007) designed a strategic sustainability evaluation framework and attempted to link reverse logistics with the TBL. Subsequently, Nikolaou et al. (2013) proposed a reverse logistics system to address corporate social responsibility and the issue of sustainability by developing a performance framework. However, several studies have pointed out that addressing sustainability does not rely only on the TBL; reverse logistics, instead of a cost minimization approach, might provide a new opportunity for manufacturing to achieve better sustainable performance (Guide and Van Wassenhove 2009; Tseng et al. 2018). Thus, future studies on the engineering aspect must take reverse logistics into account to create an appropriate approach or system for promoting efficiency and effectiveness to achieve sustainability.

## Method

**Fig. 7:**
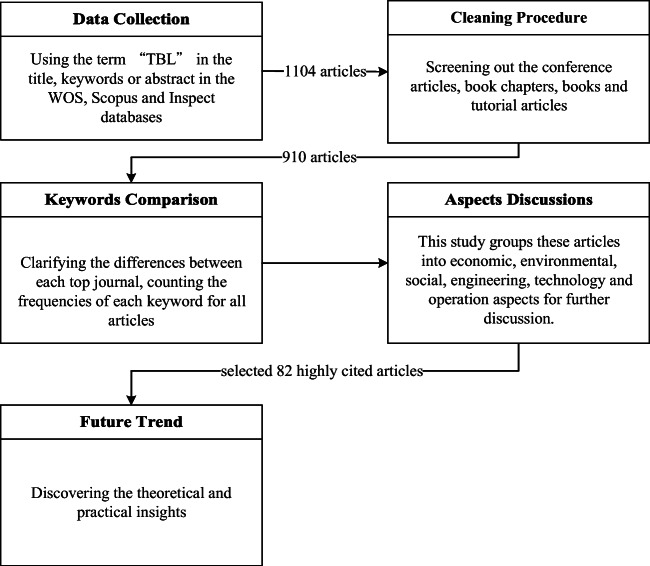
Analytical procedures of literature review

This section provides details on the data collection and cleaning process for the related articles. The analytical procedures are shown below (Fig. 7).

### Data collection

To guarantee the reliability of the literature review analysis, this study gathered articles from three major scientific research databases: Web of Science (WOS), Scopus, and Inspec. Fink (1998) emphasized that literature reviews are a systematic, explicit, and reproducible design for identifying, evaluating, and interpreting the existing body of recorded documents. Literature reviews help researchers by providing an overview of existing studies that identifies patterns, themes, and issues; reviews complete the conceptual content of the field and contribute to theory development (Harland et al. 2006; Meredith 1993; Seuring and Müller 2008; Prashar and Sunder 2019). To conduct a comprehensive analysis, the current study included articles published from January 1997 to September 2018 in a peer-reviewed journal and in the English language. The search term was “TBL” with an asterisk to ensure that the term appeared in the title, keywords, or abstract. The initial search resulted in 1104 articles related to the TBL from these three databases. To conduct the following analytical procedures, these articles were extracted to an Excel file with information on the title, total citations, keywords, abstract, journal title, abbreviated journal title, publisher, language, type of article, name(s) of the author(s), year and date of publication, categories, funding support, and countries.

### Cleaning procedure

The purpose of this procedure was to screen out conference articles, book chapters, books, and tutorial articles from the initial search results. A total of 910 articles were accepted for further analysis, including 720 articles from WOS, 132 articles from Inspec, and 58 articles from Scopus, as shown in Fig. 3. Although these articles might cover all current TBL research, reading the full content of all articles is impractical (Seuring and Müller 2008). To overcome this challenge, Martinez-Jurado and Moyano-Fuentes (2014) selected 58 articles among the hundreds of articles in their database to conduct in-depth evaluations of works on lean management, supply chain management, and sustainability. Rajeev et al. (2017) also extracted 59 articles from 1068 articles to discover the evolution period of sustainability in supply chain management. Accordingly, the current study adopts the 20 most highly cited articles to perform an in-depth discussion of each aspect.

**Fig. 8:**
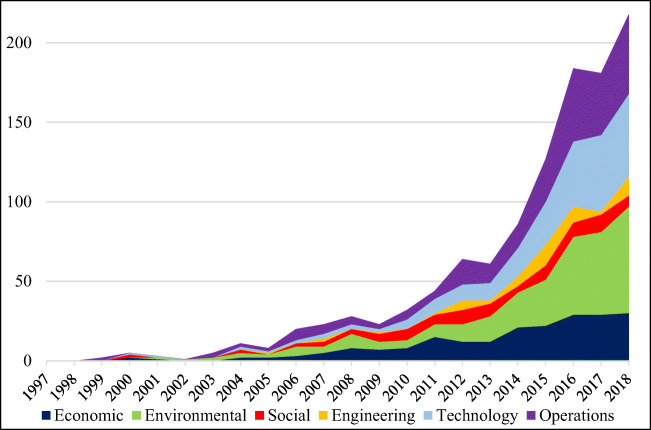
The articles published on different aspects over the past two decades

Based on the original categories, the related articles are grouped into environmental science and ecology, business economics, engineering, science and technology, computer science, social science, social issues, operations research, management science, construction building technology, and public administration. To organize these diverse perspectives, this study merges some categorizes into the six focal aspects (economic, environmental, social, engineering, technology, and operations). Although Fig. 8 shows that the number of articles on each aspect has continued to increase over the past two decades, researchers have overall focused on discussions of the environmental, technology, and operations aspects. In addition, the figure shows that the social and engineering aspects have been insufficiently discussed to support the theoretical development of the TBL over these two decades.

### Comparison of keywords

To clarify the differences among each top journal, this study counts the frequencies of each keyword for all articles. Then, wordart.com (an open source online webpage that can generate a word cloud) is applied to present the words with the highest rate of utilization in the TBL articles among different journals. In these word clouds, terms used more frequently are presented in a larger size with bold font, while terms used less frequently are displayed in a smaller font (Tseng et al. 2019). Furthermore, the top 3 published journals for each aspect are compared to explore the critical discussion over the past two decades (comparison figures are shown in Appendix A1).

#### Economic aspect

Based on the journals in which 208 articles were published, the top published journals related to the economic aspect are as follows. The *Journal of Business Ethics* (*J. Bus. Ethics*) published 18 articles (8.7%) with an impact factor of 2.917 (according to the 2017 Journal Citation Report); it represents the highest number of published articles on the business and economic issues associated with ethical considerations over the past two decades. It is followed by *Business Strategy and the Environment* (*Bus. Strateg. Environ.*), which published 14 articles (6.7%) and had the highest impact factor, 5.355 (according to the 2017 Journal Citation Report); these articles are categorized into business, environmental studies, and management, according to the WOS classification. *Supply Chain Management: An International Journal* (*Supply Chain Manag.*) ranks third; it published 9 articles (4.3%) with an impact factor of 3.833 (according to the 2017 Journal Citation Report) from the supply chain perspective that discuss economic growth while balancing the TBL.

To compare these top three journals, a word cloud comparison is employed to search for the differences by counting the frequencies of keywords from their articles. As shown in Appendix A1, the articles published in these three journals heavily feature the TBL topic as a means to solve sustainability issues. The *J. Bus. Ethics* focused on addressing the economic aspect from the corporate perspective and linking it with ethical and social considerations. *Bus. Strateg. Environ.* was concerned with issues of development and balancing performance between the economic and social aspects of business. *Supply Chain Manag.* addressed the economic aspect from the supply chain, performance, retail, and management perspectives and then linked it to the social aspect.

#### Environmental aspect

There are 292 TBL articles related to the environmental aspect, and 33.56% of these articles are published in the *Journal of Cleaner Production* (*J. Clean Prod.*), which has published 98 articles over the past two decades with an impact factor of 5.651 (according to the 2017 Journal Citation Report). Although the second highest journal, *Sustainability*, was launched only in 2009, it has published 50 articles (17.12%) over the past decade with an impact factor of 2.075. This result shows that an increasing number of researchers are willing to draw more attention to their findings by publishing in an open access journal and that they expect to become more deeply involved with sustainability projects. *Bus. Strateg. Environ.* is the third highest ranked journal with regard to the environmental aspect, with 14 articles (4.79%), while it is the second highest ranked journal with regard to the economic aspect.

More in-depth, these top 3 journals heavily feature the topic of sustainability by considering the environmental aspect of the TBL. Although all of these journals take the environmental aspect into account, there are differences in their approaches. For example, work published in the *J. Clean Prod.* uses terms such as “management,” “performance,” “supply chains,” “models,” “green,” “social,” and “industry” (as shown in Appendix A1). However, “performance,” “management,” “chain,” “supply”, “framework,” “green,” and “model” are the focal terms in the discussion on sustainability. *Bus. Strateg. Environ.* concentrates on the terms “development,” “social,” “issues,” “measurement,” “value,” “ecology,” “business,” and “corporate.” In summary, the most frequent term related to the environmental aspect in the top three journals is “performance.” Hubbard (2009) pointed out that many firms have adopted internationally recognized, industry-certified environmental management systems to tackle the challenges of measuring their environmental performance to improve their TBL balance. Cankaya and Sezen (2019) demonstrated that promoting environmental performance by adopting green supply chain practices helps firms to move toward sustainability.

#### Social aspect

Regarding the 87 published articles related to the social aspect, the top 5 journals are the *J. Bus. Ethics*, the *Journal of Sustainable Tourism* (*J. Sustain. Tour.*), *Sustainable Development* (*Sustain. Dev.*), *Business Ethics Quarterly*, and *Tourism Management*, as shown in Fig. 12*.* These journals contain 51.72% of publications on the social aspect. With 18 published articles on this domain, the *J. Bus. Ethics* is the top journal on the social aspect; it is also the top journal on the economic aspect. The *J. Sustain. Tour.* is the second highest journal; it published 13 articles with an impact factor of 3.329 (according to the 2017 Journal Citation Report). The next journal, which published 6 articles and had an impact factor of 2.750 in 2017, is *Sustain. Dev*. Notably, in this top 20 list, one-fourth of journals are related to tourism: *Tourism Management* (5th), the *Journal of Travel Research* (6th), *Current Issues in Tourism* (9th), the *Annals of Tourism Research* (17th), and the *International Journal of Tourism Research* (18th). This result shows that tourism journals are striving to fill the gaps in the TBL toward sustainability.

The terms “corporate,” “social,” “ethics,” “view,” “business,” “win,” “responsible,” “reports,” “corporate social responsibility,” “tension,” and “paradox” are repeated at least 70 times in the keywords and abstracts of articles published in the *J. Bus. Ethics*. “Business,” “tourism,” “model,” “development,” “case,” “event,” “mix,” “social,” “based,” and “hotel” are the critical terms in the articles published in the *J. Sustain. Tour*. “Stakeholders,” “development,” “accounting,” “engagement,” “urban,” “co-benefits,” “policy,” “public tourism,” and “environmental” are the most frequently repeated terms in the articles published in *Sustain. Dev.* Based on these fragmentary terms, the *J. Bus. Ethics* has strived to clarify social issues from the corporate perspective by adopting the resource-based view and the corporate social responsibility concept. The *J. Sustain. Tour.* has investigated the business and tourism events associated with a case study to develop a model with a mixed basis for addressing the social aspect of sustainability. Based on stakeholder theory and the balanced scorecard, *Sustain. Dev.* has assessed public policy and sustainability accounting to take the social aspect into account.

#### Operations aspect

The top 3 journals publishing TBL articles related to the operations aspect are the *International Journal of Production Economics* (*Int. J. Prod. Econ.*), the *International Journal of Production Research* (*Int. J. Prod. Res.*), and the *European Journal of Operational Research* (*Eur. J. Oper. Res.*), with impact factors of 4.407, 2.623, and 3.428, respectively (according to the 2017 Journal Citation Report). Here, the top journal on the operations aspect is the *Int. J. Prod. Econ.*, which published 22 articles (39.29%), integrating operations research with management science to address sustainability issues. With 11 articles (19.64%), the *Int. J. Prod. Res.* ranks second; it belongs to the same category as *Int. J. Prod. Econ.*, i.e., engineering, industry, manufacturing, operations, and management. The *Eur. J. Oper. Res.* ranks third, publishing 6 articles (10.71%). These three journals published almost 70% of the TBL articles related to the operations aspect.

The *Int. J. Prod. Econ.*, the *Int. J. Prod. Res.*, and *Eur. J. Oper. Res.* addressed the operations aspect of the TBL by considering the supply chain. In addition, the *Int. J. Prod. Econ.* concentrated on employing MCDM methods in association with fuzzy theory (such as the fuzzy analytical network process and fuzzy decision-making trials and evaluation laboratories) to assess green performance to guide management. The *Int. J. Prod. Res.* focused on developing the operational mechanism by structuring the optimal process to improve decision-making and achieve better management performance. The *Eur. J. Oper. Res.* considered the search for optimal operations through a multiobjective programming model to enhance incentives under a closed-loop supply chain.

#### Technology aspect

The situation for the technology aspect is similar to that for the environmental aspect, as the *J. Clean Prod.* and *Sustainability* are the top two journals, publishing 98 and 50 TBL articles, respectively, related to the technology aspect. The articles published in the *J. Clean Prod.* account for 41.35% of the total and those published in Sustainability account for 21.10%. In addition, an increasing number of researchers are preferring to share their research findings on the use of technology to address sustainability issues through open access journals. Finally, *J. Sustain. Tour.* ranks third, publishing 13 TBL articles (5.49%) related to the technology aspect, with an impact factor of 3.329 (according to the 2017 Journal Citation Report); this journal also ranks second with regard to the social aspect. These top 3 journals published almost 68% of all TBL articles on the technology aspect.

For a detailed comparison of the top 3 journals, word clouds are adopted, as shown in Appendix A1. The terms “management,” “supply,” “chain,” “social,” “model,” “green,” “performance,” “industry,” “environmental,” “product,” and “report” are repeated frequently in the articles published in the *J. Clean. Prod*. These terms indicate that the *J. Clean. Prod.* strives to address the technology aspect of the TBL by formulating a green model for supply chains and by developing innovation to improve the performance of management. “Supply,” “chain,” “performance,” “life,” “green,” “cycle,” “analysis,” “indicator,” “social,” “model,” “assessment,” “development,” and “water” are the frequently repeated terms in the articles published in *Sustainability*. In other words, *Sustainability* analyzes the technology aspect by adopting life cycle assessment and structuring a green model along supply chains to manage performance. *J. Sustain. Tour.* concentrates on the terms “model,” “business,” “tourism,” “development,” “case,” “event,” “economic,” “social,” “environmental,” “nature”, “mix”, “Slovenia,” “study,” and “analysis.” This result shows that the *J. Sustain. Tour.* considers tourism to be an event that should promote technological development to prevent negative impacts on nature by investigating the appropriate business model and marketing and establishing the appropriate policy. Among these terms, “performance,” “environmental,” and “social” are the terms that are common to these top 3 journals when addressing the technology aspect.

#### Engineering aspect

A total of 39.84% of related articles were published in the *J. Clean. Prod.*, 8.94% of articles were published in the *Int. J. Prod. Econ.* and 4.47% of articles were published in the *Int. J. Prod. Res*. Here, 98 articles were published in the *J. Clean. Prod.*, which means that the *J. Clean. Prod.* is a dominant journal for TBL research related to the engineering, environmental and technology aspects (these articles might overlap). The *Int. J. Prod. Econ.* ranks second, with 22 published articles. Eleven articles were published in the third highest ranking journal, the *Int. J. Prod. Res*. These journals have published 53.25% of related articles on the engineering aspect.

The word cloud for the *J. Clean. Prod.* indicates that “management,” “supply,” “chain,” “performance,” “model,” “green,” “social,” “product,” “business,” “oil,” “life environmental,” “gas,” “system,” “report,” and “decision” are the highly repeated terms in the articles. For the articles published in the *Int. J. Prod. Econ.*, the frequently repeating terms include “supply,” “chain,” “social,” “multi,” “green,” “fuzzy,” “performance,” “decision,” “theory,” “design,” “supplier,” and “environmental.” Finally, “supply,” “chain,” “multi,” “performance,” “green,” “management,” “decision,” “process,” “environmental,” “co,” and “supplier” are the repeated terms that appear in the *Int. J. Prod. Res*. In other words, these three journals take the engineering aspect of the TBL into account by addressing the issue of green supply chains and the management performance. However, there are still differences in the pursuit of sustainability. For example, the articles published in the *J. Clean. Prod.* paid more attention to employing evaluation models (such as life cycle assessment and the analytical network process) to achieve lean production and green suppliers. The articles published in the *Int. J. Prod. Econ.* preferred to utilize MCDM methods in association with fuzzy theory to explore decisive sustainable practices. In the *Int. J. Prod. Res.*, the published articles emphasized reducing costs and promoting green manufacturing by improving the efficiency of reverse logistics and optimizing the procurement mechanism.

Although these terms represent the focal area of the journals under consideration, there is still an overlap between the technology and the engineering aspects. To provide a clear boundary between these two aspects, this article provides a definition of the engineering aspect of the TBL as “adopting the creative application of science uses for designing and generating the appropriate tools or systems for ensuring economic growth, preventing negative impacts on the environment and fulfilling public expectations regarding sustainability.”

### Descriptive analysis

Based on the previous discussion, it can be seen that many researchers have investigated the relationships between the TBL, sustainability, and performance. In the beginning stage, the TBL was widely accepted to address sustainability in terms of the economy, the environment, and society (Sridhar and Jones 2013). However, several articles have shown that firms are able to generate benefits by addressing environmental and social issues (Dentchev 2004; Husted and de Jesus Salazar 2006). Carter and Easton (2011) emphasized that achieving sustainability necessarily involves falling into the intersection of the TBL. Based on a summary of the six aspects addressed above, the analytical results demonstrate that achieving sustainability by balancing the economic, environmental, and social aspects is no longer sufficient; support from operations, technology and engineering must be obtained. Some articles have focused on exploring effective ways to develop sustainability by shifting the TBL concept to the operational level (Pagell and Gobeli 2009; Gimenez et al. 2012; Shi et al. 2017). If firms are unable to consider aspects beyond the TBL, then they may gradually begin to ignore the requirements of policy, which will generate negative effects and can even threaten human life (Wu et al. 2018).

**Fig. 9:**
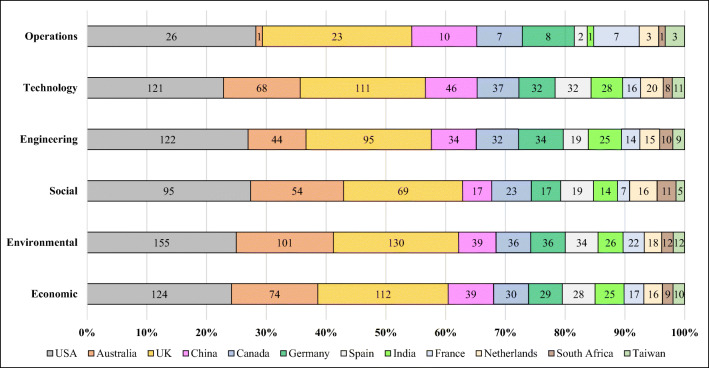
Top 12 countries/areas publishing TBL articles on different aspects over the past two decades

Based on Fig. 9, the USA, Australia, the UK, and China are the top 4 countries with researchers addressing this topic, publishing almost 65% of all TBL articles. Here, developed countries have better performance than developing countries in discussing the TBL. Rajeev et al. (2017) discovered a similar result and stated that developed economies were more mature than those of emerging markets for launching sustainable development. Thus, developing countries need to attempt to explore the appropriate approaches or systems to help them develop sustainability. For example, China has proposed the Belt and Road Initiative in association with the TBL concept to enhance economic growth, to prevent negative impacts on the environment, and to attempt to promote public awareness of sustainability (Cui et al. 2018). Furthermore, conducting a sustainable campaign is not just the duty of an individual country; it relies on all countries to share their experiences and knowledge in launching sustainable development. Thus, this article not only provides a comprehensive literature review covering the past two decades but also summarizes the approaches, theories, and gaps to guide future studies in successfully dealing with sustainability issues.

**Fig. 10:**
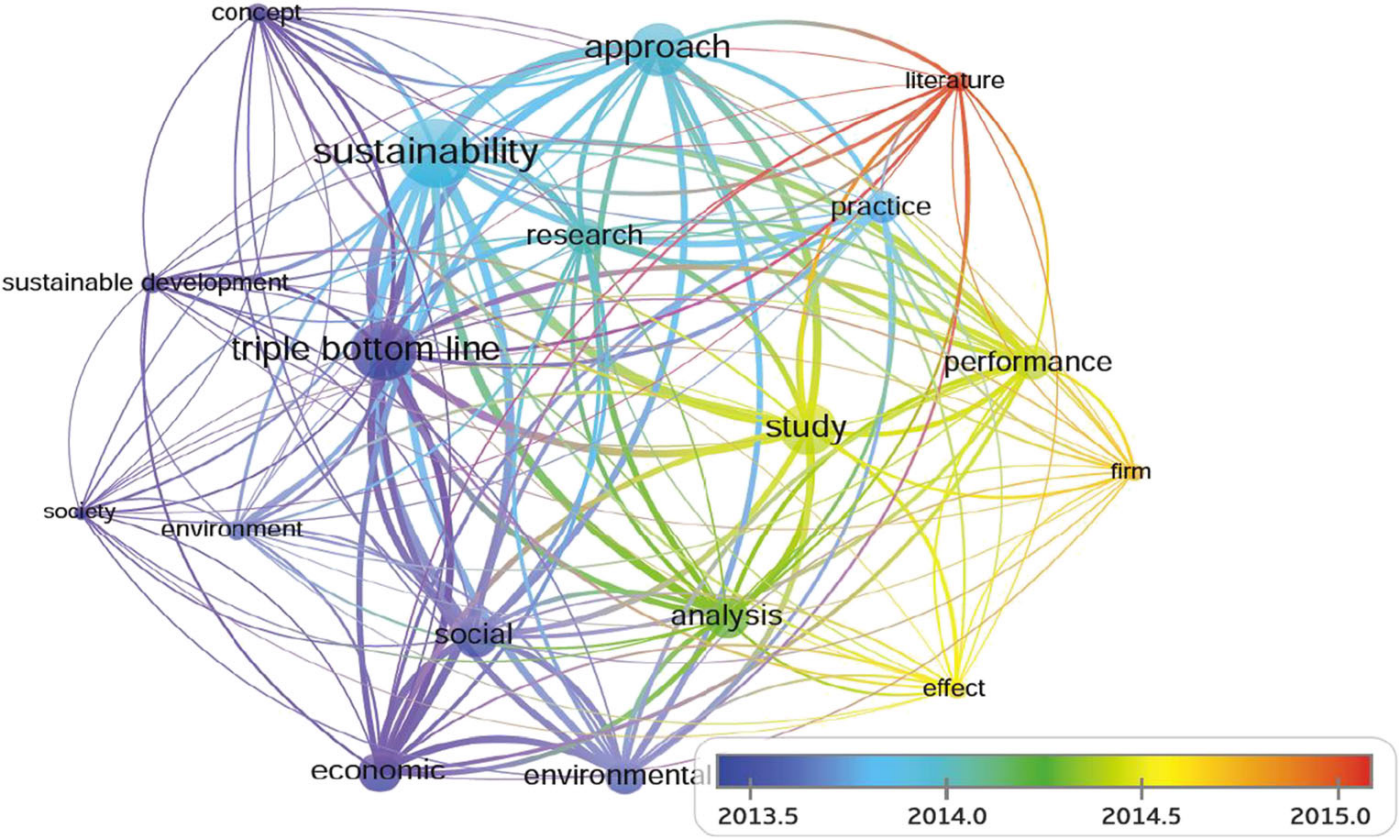
The network diagram of keywords and abstracts for all TBL articles (with a co-occurrence rate of at least 70 times)

To explore the research trends of the TBL, this study applies VOSviewer software, which is a free software package for constructing and visualizing bibliometric networks, as shown in Fig. 10. This software enables the presentation of relationships in graphical bibliometric maps (Eck and Waltman 2010). In this study, the rate of co-occurrence is set to at least 70 times between the keywords and abstracts to track research trends based on all TBL articles. It is shown that in the early stage, discussions on the TBL focused on the TBL concept by identifying the relationships between the economic, environmental, and social aspects for launching sustainable development. Then, in the following stage, the discussions shifted to exploring the appropriate approaches and practices for developing sustainability. In the recent stage, the focal points have shifted to case study analysis to check the effects on improving performance. Additionally, in recent years, comprehensive literature reviews have become popular for identifying the gaps among theories, methods and practices.

## The insights from 82 highly cited articles

This section summarizes the 82 highly cited articles from the previous discussion of the six aspects of the TBL to discover the existing gaps and future trends and reinforce the theoretical and practical insights for TBL. This summary provides a clear picture of the TBL, allowing future studies to capture the concept easily and extensively.

### Theoretical insights

In Table 1, these significant articles are arranged by year. In this table, the articles are presented specifically with respect to their differences based on the data sources, theories applied, methods adopted, and types of contributions to provide comprehensive theoretical insights for developing the TBL.

**Table 1:** Summarizing 82 highly cited articles from the previous discussions

Author(s)	Citations	Data sources	Theories applied	Methods adopted					Contributions			
				Empirical study	MCDM	LCA	Algorithm	Review	Theoretical	Managerial	Methodological	Policy
Spiller (2000)	62	Questionnaire	Business ethics					√	√	√		
Bordass et al. (2001)	84	Questionnaire	SD					√	√			√
Azapagic (2003)	97	Questionnaire	SD, CSR					√			√	
Font and Harris (2004)	78	Interview, questionnaire	CSR					√	√			
Norman and MacDonald (2004)	250	No data	ST					√	√	√		
Pope et al. (2004)	359	No data	Environmental assessment					√	√			
Foran et al. (2005)	87	Secondary data	SCM			√			√	√		
Kleindorfer et al. (2005)	579	No data	SCM					√	√			
Hatt et al. (2006)	85	Secondary data	Stormwater recycling					√	√			
Northcote and Macbeth (2006)	48	Questionnaire	STD					√	√	√		√
Dixon and Clifford (2007)	86	Interview	Entrepreneurship theory	√						√		√
Pava (2007)	27	No data	SD					√	√			
Presley et al. (2007)	77	Secondary data	CSR					√	√			√
Sayce et al. (2007)	58	Questionnaire	Sustainable property					√	√	√		√
Schianetz et al. (2007)	62	No data	Organizational learning theory					√	√	√		
Allwood et al. (2008)	41	Secondary data	SD					√	√	√		
Carter and Rogers (2008)	846	Secondary data	Resource dependence theory, RBV					√	√	√		
Solomon et al. (2008)	68	No data	Resource policy, community relations					√	√			√
Goerner et al. (2009)	65	Experimental data	SD				√		√		√	
Hubbard (2009)	178	Secondary data	ST	√					√	√		
Kleine and Hauff (2009)	51	No data	CSR, CS					√	√			
Pagell and Gobeli (2009)	88	Interview, secondary data	RBV	√					√	√		
Timur and Getz (2009)	36	Interview, questionnaire	SD, ST	√					√	√		√
Wiedmann et al. (2009)	86	Secondary data	CS				√		√	√		
Chen et al. (2010)	101	Questionnaire	ST	√						√		
Darcy et al. (2010)	41	Interview	STD, SD					√	√			
Menz (2010)	44	Questionnaire	CSR	√					√	√		
Schaltegger and Burritt (2010)	111	Questionnaire	ST					√	√			
Skouloudis et al. (2010)	40	Questionnaire	CSR, SD					√	√			
Tate et al. (2010)	192	Secondary data	ST	√						√		
Carter and Easton (2011)	368	Secondary data	ST, RBV					√	√	√	√	
Chabowski et al. (2011)	107	Secondary data	Social network theory					√	√			
Cornelissen et al. (2011)	27	No data	SD					√	√	√		√
Cronin et al. (2011)	151	Secondary data	ST					√	√			
Dao et al. (2011)	125	No data	RBV					√	√	√		
Freeman and Hasnaoui (2011)	57	Secondary data	CSR					√		√		√
Meehan and Bryde (2011)	63	Questionnaire	SD, green procurement					√	√	√		√
Reza et al. (2011)	56	Interview	Sustainable construction		√	√			√	√		
Romijn and Caniels (2011)	40	Secondary data	Evolutionary theory, SD					√				√
Wu and Pagell (2011)	210	Interview	ST	√					√	√		
Assaf et al. (2012)	33	Questionnaire	SD	√						√	√	
Dai and Blackhurst (2012)	60	Questionnaire	SCM, SD		√				√	√		
Gibson et al. (2012)	50	Secondary data	STD					√		√		
Gimenez et al. (2012)	174	Secondary data	RBV	√					√	√		
Gond et al. (2012)	89	No data	Strategic management theory					√	√			
Gopalakrishnan et al. (2012)	65	No data	CSR, SD					√	√	√		√
Hollos et al. (2012)	87	Questionnaire	RBV	√							√	
Klassen and Vereecke (2012)	127	Interview, secondary data	ST, CSR	√					√	√		
Lee et al. (2012)	65	No data	Product service system, SD					√	√	√		
Mori and Christodoulou (2012)	147	No data	Ecological perspective					√	√			√
Prajogo et al. (2012)	49	Questionnaire	SD	√					√	√		√
Roca and Searcy (2012)	122	Secondary data	CSR, SD, legitimacy theory					√	√	√		
Sarkis et al. (2012)	40	Questionnaire, secondary data	SD, SCM		√				√	√		
Shahriar et al. (2012)	61	Questionnaire	Risk assessment, SD				√		√	√		
Akadiri et al. (2013)	69	Questionnaire	Fuzzy set theory		√				√	√	√	
Gleim et al. (2013)	111	Secondary data	Social dilemma theory						√	√		
Govindan et al. (2013)	223	Questionnaire	ST		√					√	√	
Hahn and Kuehnen (2013)	152	Secondary data	ST, IT, legitimacy theory					√	√			
Lai et al. (2013)	33	Questionnaire	SD, SCM	√					√	√		
Milne and Gray (2013)	164	No data	ST					√	√	√		
Nikolaou et al. (2013)	75	Secondary data	SCM, CSR	√					√	√	√	
Winter and Knemeyer (2013)	99	Secondary data	SCM					√	√	√		
Wu (2013)	268	No data	Maslow’s hierarchy of needs, SD					√	√			
Zhu et al. (2013)	158	Interview	IT	√					√	√		√
Beske and Seuring (2014)	81	No data	Risk management, SCM					√	√	√		
Bocken et al. (2014)	343	Questionnaire	ST					√	√		√	
De Giovanni and Zaccour (2014)	44	Experimental data	SCM				√		√	√		
Devika et al. (2014)	98	Experimental data	SCM, CSR				√		√	√		
Govindan et al. (2014)	119	Experimental data	SCM				√			√		
Ji et al. (2014)	28	No data	SD					√	√	√		
Klewitz and Hansen (2014)	156	Secondary data	RBV					√	√		√	
Stylidis et al. (2014)	66	Questionnaire	SD, STD					√	√	√		
Brandenburg and Rebs (2015)	37	Secondary data	SCM, CSR					√	√	√		√
Govindan et al. (2015)	39	Experimental data	SD, SCM				√		√	√		
Hahn et al. (2015)	92	No data	CS					√	√	√	√	
Mori and Yamashita (2015)	27	No data	SD					√	√			
Sarkis and Dhavale (2015)	52	Secondary data	SD				√		√	√		
Taticchi et al. (2015)	37	Secondary data	SD, SCM					√	√	√		
Wu et al. (2015)	44	Interview, questionnaire	Fuzzy set theory, SCM		√				√	√		√
Jarvis et al. (2016)	27	Questionnaire, secondary data	SD	√					√	√		
Wilhelm et al. (2016)	38	Questionnaire	SCM, agency theory					√	√	√		
Dubey et al. (2017)	42	Questionnaire	SCM					√	√	√		
Note: Corporate sustainability (CS), corporate social responsibility (CSR), institutional theory (IT), resource-based view (RBV), supply chain management (SCM), sustainable development (SD), stakeholder theory (ST), sustainable tourism development (STD)												

#### Data source

**Fig. 11:**
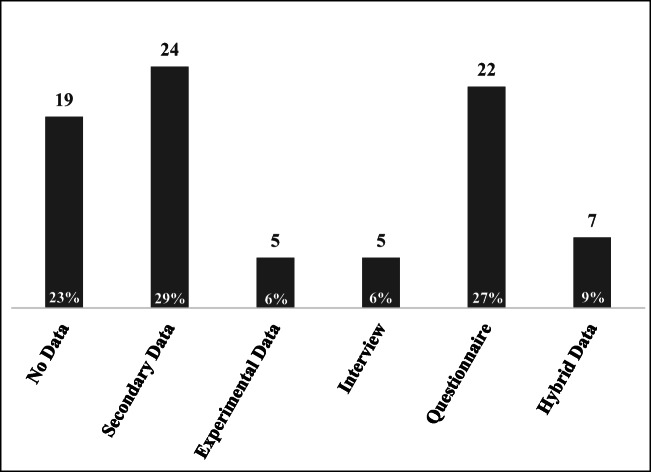
Total data sources from summarizing 82 articles

Figure 11 presents the different kinds of data sources employed in these selected articles. Here, 23% of the articles have no data and focus on developing a conceptual framework. For instance, Pope et al. (2004) proposed an alternative concept of sustainability assessment and provided a clarification of different approaches described in the literature as forms of sustainability assessment. Hubbard (2009) conducted stakeholder-based analysis in association with a sustainable balanced scorecard conceptual framework to assess organizational sustainability performance. To overcome this shortage of primary data, 29% of articles have attempted to employ secondary data to address the TBL issue. Here, Tate et al. (2010) utilized content analysis by assessing the information from 10 corporate social responsibility reports, which included 6500 pages. Klewitz and Hansen (2014) employed a systematic review to summarize different types of sustainability-oriented innovation for small- and medium-sized enterprises over the 1987–2010 period. Furthermore, 5 articles have attempted to address the TBL issue by employing experimental data. For example, Devika et al. (2014) developed a multiobjective model for identifying the impacts of the TBL by integrating imperialist competitive algorithms with variable neighborhood search. Govindan et al. (2014) used a two-echelon location-routing problem with time windows to solve the problem of a perishable supply chain, optimizing the TBL objectives using multiobjective particle swarm optimization and multiobjective variable neighborhood search. However, the issue with respect to the TBL is becoming more complex. Thus, 7 articles have tried to use hybrid data. Pagell and Gobeli (2009) combined qualitative and quantitative data to examine the relationship between operational managers’ experiences with and attitudes toward employee well-being and then to identify the effects with regard to the TBL. Jarvis et al. (2016) formulated a questionnaire in association with secondary data to discover the decisive factors affecting the TBL to assess tourist satisfaction and forecast potential tourist return rate. Because the issue is becoming more complex and uncertain, combining data to carry out comprehensive considerations and to overcome uncertainty can be the next stage toward which future research can strive.

#### Theories applied

**Fig. 12:**
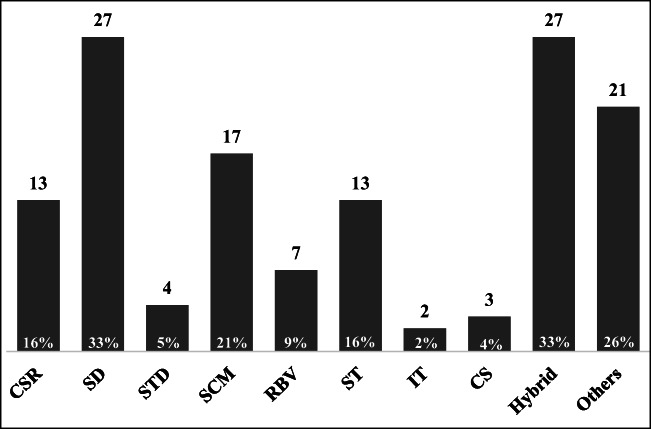
Frequencies of the theories applied from summarizing 82 articles (the frequencies overlap)

To address the TBL issue with a mature approach, studies have applied certain theories, including the resource-based view, stakeholder theory, and sustainable development. However, the real conditions and issues are changing rapidly and becoming more complex over time. If an article wants to address these issues through a single theory, doing so is becoming increasingly difficult. As shown in Fig. 12, 33% of articles employed sustainable development and hybrid theories. Here, Azapagic (2003) considered sustainable development and corporate social responsibility and developed a corporate sustainability management system to guide businesses in becoming sustainable by providing systematic, step-by-step guidelines. Moreover, Wu (2013) combined Maslow’s hierarchy of needs with sustainable development to develop a framework of sustainability. Additionally, 26% of articles have attempted to apply other theories to provide a different perspective to address the TBL. For example, Roca and Searcy (2012) and Hahn and Kuehnen (2013) tried to address the TBL by employing the legitimacy theory. A total of 21% of articles integrate the supply chain management concept into the TBL to achieve sustainability. Corporate sustainability and stakeholder theory are utilized in approximately 16% of articles. Although several individual theories and hybrid theories have been well discussed to promote a better understanding of the TBL from diverse considerations, an extensive theoretical framework for bridging theories and practices is still lacking.

#### Methods adopted

**Fig. 13:**
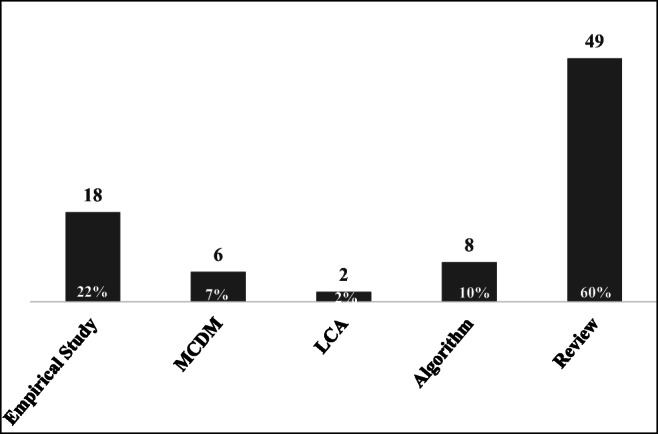
Frequencies of the methods adopted from summarizing 82 articles (the frequencies overlap)

With regard to the methods of these 82 articles, 60% of articles implemented a review method to explore the theoretical basis of the TBL (as shown in Fig. 13). These articles attempted to address sustainability issues by integrating the TBL concept and offering a solid basis for developing theory. For example, Carter and Rogers (2008) conducted a large-scale literature review to extend the TBL concept from people, the planet, and profits to economic, environmental, and social considerations. Next, 21% of articles applied empirical research to examine the relationships among the proposed aspects; in this respect, these articles generally adopted questionnaires and hypothesis testing. Additionally, exploratory factor analysis and confirmatory factor analysis were employed to confirm the validity of the proposed aspects. In these 82 articles, only 7% utilized MCDM methods to balance the TBL. Here, Akadiri et al. (2013) proposed a building material selection model associated with a fuzzy extended analytical hierarchy process. Govindan et al. (2013) applied the fuzzy TOPSIS method to develop an effective model for sustainable supply chain initiatives considering the TBL. Foran et al. (2005) and Reza et al. (2011) represent two rare articles that adopted life cycle assessment for their TBL discussions. Foran et al. (2005) proposed a framework integrating sustainable supply chain management with TBL accounting by utilizing life cycle assessment based on input-output analysis. Based on these efforts, future studies must strive to improve quantitative analysis methods to offer precise figures to guide practitioners in achieving sustainability by adopting the TBL. Moreover, there is an essential need to integrate qualitative and quantitative analysis to conduct a comprehensive discussion.

#### Types of contributions

**Fig. 14:**
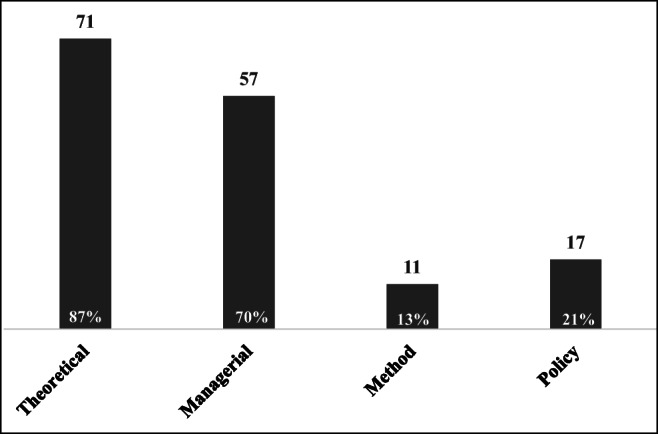
Total frequencies of contributions from summarizing 82 articles (the frequencies overlap)

From summarizing the articles, four types of contributions can be categorized, i.e., theoretical, managerial, methodological, and policy contributions, to provide directions for future research. As shown in Fig. 14, 87% of the articles strived to provide a theoretical basis to strengthen and enhance understanding of the TBL. Spiller (2000) established a new integrated model for ethical businesses and investors to achieve TBL performance. Approximately 70% of the articles generated managerial contributions to guide firms in balancing the TBL. For instance, Gleim et al. (2013) identified numerous barriers to sustainability accounting to offer precise practices for firms to gain green market share based on TBL considerations. Among these articles, only 13% provided methodological contributions. In particular, Goerner et al. (2009) combined thermodynamic, network, and information theoretic measures with real-time ecosystems to obtain assessment results and related concepts, providing a novel method for evaluating long-term economic health and sustainability. Twenty-one percent of articles generated policy contributions. For example, Timur and Getz (2009) proposed that government agencies should play an important role in developing sustainable tourism because they are responsible for setting up industrial plants, establishing policies and regulations, and monitoring implementations to guide firms in achieving the TBL.

### Practical insights

This section summarizes these 82 significant articles from the industrial view to identify a direction for balancing the TBL. Moreover, this study attempts to bridge the gap between theory and practice to provide precise guidelines.

#### Industrial classifications and existing arguments

**Fig. 15:**
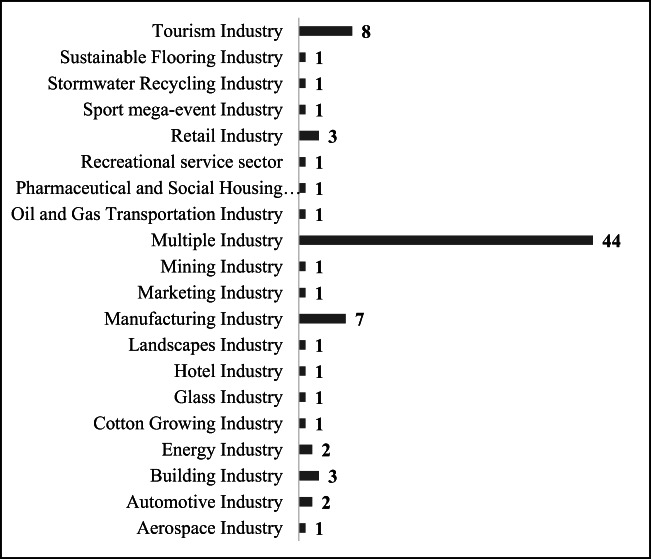
Total frequencies of industrial classification from summarizing 82 articles

As shown in Fig. 15, the 82 articles were classified as covering 20 industries. Within these articles, almost 54% of the discussion was related to multiple industries. These articles focused on supply chain, supplier selection, performance assessment, corporate social sustainability, management style, business models, information technology, and operations. For example, Cater and Easton (2011) investigated several industries, including the automotive sales industry, the consumer products industry, the food and beverage industry, and the transportation industry, to assess the TBL. Govindan et al. (2013) focused on considering the TBL aspect when selecting suppliers and proposed simultaneously taking sustainable business operation into account during selection. Nikolaou et al. (2013) indicated that sustainable reverse logistics should not only be related to environmental and financial performance but also be required to consider social performance in the measurement of a firm’s sustainability performance.

The second most discussed industry is the tourism industry, which is the focus of 8 articles (accounting for 10%). Darcy et al. (2010) performed a case study to realize sustainability targets by balancing the TBL and argued that tourism operators need to use the broader concept of the TBL in relation to business activities. Furthermore, these 8 articles focused on structuring a conceptual framework for sustainable tourism development. The manufacturing industry is the third most highly discussed industry, with four articles (accounting for almost 8.5%) focusing on this industry. For example, Lai et al. (2013) tried to clarify whether reverse logistic practices enable Chinese manufacturers to develop a sustainable competitive advantage to enhance their environmental and financial performance through recycling, reusing, reprocessing, and designing. Among these four articles, Lai et al. (2013), Zhu et al. (2013), and Dubey et al. (2017) addressed manufacturing issues related to TBL concerning the supply chain, and Gimenez et al. (2012) addressed operations issues.

#### Arranging aspects

Table 2 shows that the 82 articles are categorized into six aspects, as discussed in the previous section. These articles demonstrate that to address sustainability issues, at least two aspects of the TBL must be balanced. This evidence supports the argument that the traditional aspects of the TBL are insufficient to cover the entire concept of sustainability (Wu et al. 2016). To provide a better illustration, the TBL should take the technology, engineering, and operations aspects into account. Moreover, these aspects are interrelated with real practices, and further studies must consider this interrelationship when dealing with sustainability issues. In this table, 8 articles addressed sustainability issues by covering the environment, technology, and engineering aspects. This table reveals that the majority of articles addressed environmental issues by considering the technology and engineering aspects in advance (Govindan et al. 2013; Bocken et al. 2014; Nikolaou et al. 2013) and that few studies perfectly balanced the economic, environmental, and social aspects. Thus, balancing the TBL toward sustainability not only relies on enhancing economic growth, preventing negative impacts on the environment, and fulfilling social expectations but also requires support from technology, engineering, and operations to promote efficiency and effectiveness among the practices.

**Table 2:** Arranging aspects from summarizing 82 articles

Aspects articles	Social	Economic	Environmental	Technology	Engineering	Operations
Spiller (2000)	√					
Bordass et al. (2001)				√		
Azapagic (2003)					√	
Font and Harris (2004)	√					
Norman and MacDonald (2004)	√	√				
Pope et al. (2004)			√			
Foran et al. (2005)		√	√			
Kleindorfer et al. (2005)					√	√
Hatt et al. (2006)			√			
Northcote and Macbeth (2006)	√					
Dixon and Clifford (2007)		√				
Pava (2007)	√					
Presley et al. (2007)					√	√
Sayce et al. (2007)				√		
Schianetz et al. (2007)	√		√			
Allwood et al. (2008)				√		
Carter and Rogers (2008)		√				
Solomon et al. (2008)			√			
Goerner et al. (2009)			√			
Hubbard (2009)		√	√			
Kleine and Hauff (2009)	√					
Pagell and Gobeli (2009)					√	√
Timur and Getz (2009)	√					
Wiedmann et al. (2009)			√	√	√	
Chen et al. (2010)					√	√
Darcy et al. (2010)	√			√		
Menz (2010)	√					
Schaltegger and Burritt (2010)		√				
Skouloudis et al. (2010)				√		
Tate et al. (2010)		√				
Carter and Easton (2011)		√				
Chabowski et al. (2011)		√				
Cornelissen et al. (2011)	√					
Cronin et al. (2011)		√				
Dao et al. (2011)	√			√		
Freeman and Hasnaoui (2011)	√					
Meehan and Bryde (2011)			√			
Reza et al. (2011)				√		
Romijn and Caniels (2011)	√					
Wu and Pagell (2011)		√				√
Assaf et al. (2012)	√					
Dai and Blackhurst (2012)						√
Gibson et al. (2012)	√					
Gimenez et al. (2012)					√	√
Gond et al. (2012)		√				
Gopalakrishnan et al. (2012)					√	√
Hollos et al. (2012)					√	√
Klassen and Vereecke (2012)					√	√
Lee et al. (2012)			√	√	√	
Mori and Christodoulou (2012)			√			
Prajogo et al. (2012)			√	√		
Roca and Searcy (2012)			√	√	√	
Sarkis et al. (2012)				√		
Shahriar et al. (2012)					√	
Akadiri et al. (2013)				√	√	
Gleim et al. (2013)		√				
Govindan et al. (2013)			√	√	√	
Hahn and Kuehnen (2013)			√	√	√	
Lai et al. (2013)						√
Milne and Gray (2013)	√	√				
Nikolaou et al. (2013)			√	√	√	
Winter and Knemeyer (2013)		√				
Wu (2013)			√			
Zhu et al. (2013)		√				
Beske and Seuring (2014)		√				
Bocken et al. (2014)			√	√	√	
De Giovanni and Zaccour (2014)						√
Devika et al. (2014)		√				√
Govindan et al. (2014)					√	√
Ji et al. (2014)						√
Klewitz and Hansen (2014)			√	√	√	
Stylidis et al. (2014)	√		√			
Brandenburg and Rebs (2015)						√
Govindan et al. (2015)						√
Hahn et al. (2015)	√	√				
Mori and Yamashita (2015)	√					
Sarkis and Dhavale (2015)						√
Taticchi et al. (2015)						√
Wu et al. (2015)						√
Jarvis et al. (2016)	√					
Wilhelm et al. (2016)						√
Dubey et al. (2017)				√		

#### Cross discussion between industrial classifications and arranged aspects

To bridge the gap between theory and practice, this article utilizes a cross table to present the industrial classifications considering the aspects discussed in the previous section. Table 3 shows that for 7 industries, there are insufficient studies addressing sustainability issues, which means that other aspects must be taken into account to facilitate further discussion. The hotel and sport mega-event industries concentrate on social aspects. The sustainable flooring industry has to pay much attention to developing technology to pursue sustainability. The landscaping, pharmaceutical, mining, and stormwater recycling industries strive to prevent environmental impacts.

**Table 3:** Cross table between industrial classifications and arranged aspects

	Aspects					
Industrial classifications	Social	Economic	Environmental	Technology	Engineering	Operations
Aerospace industry					√	√
Automobile industry	√	√				√
Energy industry	v			√		
Building industry				√	√	√
Cotton growing industry		√	√			
Glass industry		√				√
Hotel industry	√					
Landscapes industry			√			
Manufacturing industry		√	√	√	√	√
Marketing industry					√	√
Mining industry			√			
Multiple industries	√	√	√	√	√	√
Oil and gas transportation industry					√	
Pharmaceutical and social housing industry			√			
Recreational service industry			√	√	√	
Retail industry		√				√
Sport mega-event industry	√					
Stormwater recycling industry			√			
Sustainable flooring industry				√		
Tourism industry	√		√			

Several industries have attempted to cross two aspects to address sustainability issues and enhance the success of improving sustainability. These industries include aerospace and marketing, which cross the engineering and operations aspects. Gopalakrishnan et al. (2012) pointed out that a lack of environmental mandates from the aerospace industry is obviously a drawback and argued that an integrated supply chain with sustainability objectives for the aerospace industry is key to ensuring a supply chain-wide economic, social, and environmental balance. The glass and retail industries take economic and operations aspects into account. The automotive sales, cotton growth, and tourism industries focus on socioeconomic and environmental aspects to tackle sustainability issues.

Although four industries take three aspects into account to improve sustainability performance, there still remain gaps on the path to sustainability. For instance, the recreational service industry addresses sustainability issues by considering environmental, technology and engineering aspects, but economic and social aspects also generate impacts (Manzini and Vezzoli 2002; Lee et al. 2012). In addition, the aspects of technology, engineering, and operations are adopted by the building industry.

## Conclusions

In 1997, Elkington proposed the concept of a TBL comprising people, the planet and profits to address the issue of sustainability. Carter and Rogers (2008) extended this concept to the economy, the environment, and society. However, Wu et al. (2016) argued that these three aspects are insufficient if the TBL is to cover the entire concept of sustainability. To overcome this issue, this study conducts an extensive literature review on the TBL by employing a bibliometric analysis of articles published from January 1997 to September 2018. Subsequently, 910 related articles from three major scientific research databases, i.e., WOS, Scopus, and Inspec, were screened. To obtain detailed information and insights, word clouds are employed to compare the highest frequencies of terms among different journals to present their features. Network analysis is adopted to display the research and authorship trends over the past two decades. Moreover, the 82 most highly cited articles on each aspect are selected for further discussion.

The results provide solid evidence indicating that the traditional TBL is insufficient to cover the entire concept of sustainability: engineering, technology, and operations aspects must also be taken into account. Over the past two decades, studies have placed too much emphasis on discussing environmental, technology, and operations aspects. Although interest in the social and engineering aspects is increasing annually, these incremental discussions are still unable to address other aspects. The USA, Australia, the UK, and China accounted for more than 60% of the TBL articles on each aspect. However, only two Asian universities, Dalian University of Technology and Hong Kong Polytechnic University, have attempted to discuss the TBL over the past two decades. Based on these related articles, discussions on the TBL have shifted from sustainability to micro considerations in terms of firm performance assessment and effect investigation.

Summarizing the 82 articles, most highly cited articles on each aspect reveals that secondary data and questionnaires are the two main data sources used by the articles to address the TBL. Although corporate sustainability, corporate social responsibility, institutional theory, the resource-based view, supply chain management, sustainable development, stakeholder theory, and sustainable tourism development have been applied to address the TBL, approximately 30% of articles have attempted to discover an appropriate theoretical basis for supporting the TBL by utilizing hybrid theories and others. Regarding the methods adopted, almost 60% of articles selected a literature review as the method for discovering the precise framework for the TBL. Additionally, 22% of articles adopted empirical research to explore the decisive factors of the TBL. Among these articles, 87% strived to contribute to the theoretical basis and 70% of articles provided managerial contributions. Future studies need to strive to employ hybrid methods and hybrid data to reinforce the theoretical basis of the TBL and provide policy and methodological contributions because the real problem is becoming increasingly complex.

The 82 summarized articles cover 20 industrial classifications. Of these articles, 44 (accounting for almost 54%) addressed TBL issues related to multiple industries. The tourism industry is the second most highly discussed industry, with 8 articles (approximately 10%); however, these articles addressed sustainability issues by considering social and environmental aspects only. The third most highly discussed industry is the manufacturing industry, which includes 7 articles (approximately 8.5%) that consider balanced economic, engineering, and operations aspects to achieve sustainability. These 20 industries attempted to tackle the sustainability issue by considering environmental, engineering, and operations, in contrast to previous articles focused on achieving sustainability by balancing economic, environmental, and social aspects. This finding confirms another gap between theory and practice. To bridge this gap, further articles need to consider other aspects to enhance the efficiency and effectiveness of improvements. Currently, only 4 industries have covered more than three aspects to increase the success of sustainable development. Industrial practitioners and researchers must balance the proposed six aspects simultaneously and urgently to facilitate global sustainability.

Although the bibliometric analysis provided in this article attempts to cover as many published words as possible, there are still several limitations. This article focused only on studies published in English, and several articles that were published in languages other than English were excluded from the selection. Future studies must integrate these non-English-language articles in their considerations to offer a more generalized discussion. In addition, this article focuses on only three major databases, WOS, Scopus, and Inspec, to select the related articles. Future studies can attempt to include the Google Scholar database to conduct a more comprehensive literature review. This article adopts basic statistics to present the results, and future studies may utilize content analysis for each related article to gather more in-depth information to support their theoretical basis.

## Electronic supplementary material

ESM 1 (DOCX 1719 kb)
