# Supplementary material for: Future trends and guidance for the triple bottom line and sustainability: a data driven bibliometric analysis
Source: Environ Sci Pollut Res Int. 2020 Jun 22;27(27):33543–67. doi: 10.1007/s11356-020-09284-0 (PMC7423869; doi:10.1007/s11356-020-09284-0)
Supplement: Supplementary file 1 — (DOCX 1719 kb) [file 11356_2020_9284_MOESM1_ESM.docx]

Appendix

A1. Top 3 journal comparisons for each aspect


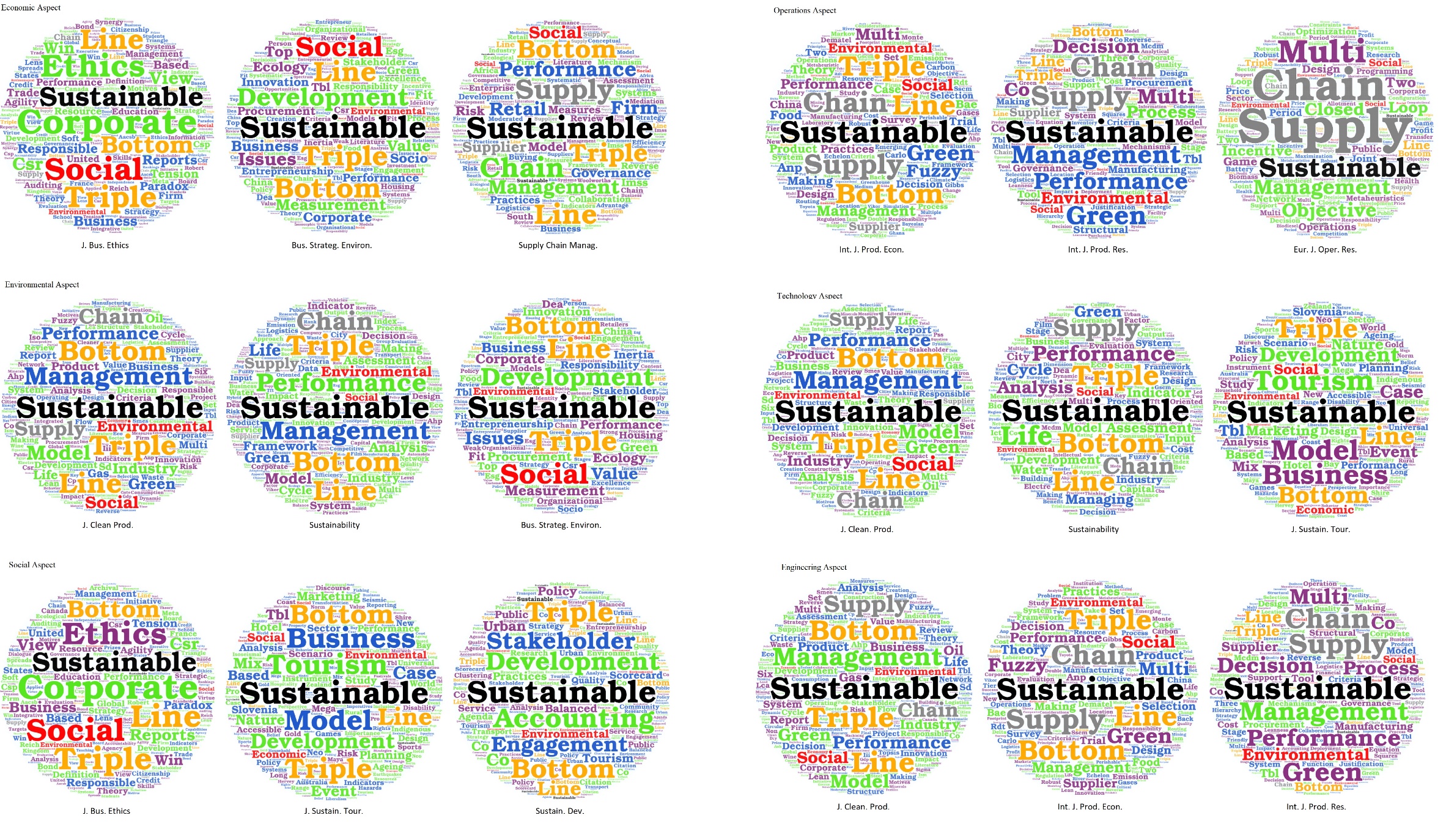


A2. The 20 Most Highly Cited TBL Articles on the Economic Aspect

| References | Citations | Journal | Summary |
| --- | --- | --- | --- |
| Carter & Rogers (2008) | 846 | Int. J. Phys. Distrib. Logist. Manag. | The authors perform a large-scale literature review and use conceptual theory building to introduce the concept of sustainability to the field of supply chain management and to demonstrate the relationships between environmental, social, and economic performance in a supply chain management context. |
| Carter & Easton (2011) | 368 | Int. J. Phys. Distrib. Logist. Manag. | The article provides a valid review of the evolution of empirical sustainable supply chain management research across a 20‐year time period. It finds that the field of sustainable supply chain management has evolved from a perspective and investigation of standalone research in social and environmental areas to the beginning of the convergence of sustainability perspectives through the TBL and the emergence of sustainable supply chain management as a theoretical framework. |
| Norman & MacDonald (2004) | 250 | Bus. Ethics Q. | This paper argues on both conceptual and practical grounds that the TBL is an unhelpful addition to current discussions of corporate social responsibility. |
| Wu & Pagell (2011) | 210 | J. Oper. Manag. | In this study, theory is built through case studies to answer the following question: how do organizations balance short-term profitability and long-term environmental sustainability when making supply chain decisions under conditions of uncertainty? |
| Tate et al. (2010) | 192 | J. Supply Chain Manag. | This research uses content analysis software to examine corporate communications to stakeholders through corporate social responsibility reports in a context where firms are increasingly under pressure from stakeholders to incorporate the TBL comprising social, environmental and economic responsibility considerations into operations and supply chain management strategies. |
| Hubbard (2009) | 178 | Bus. Strateg. Environ. | This study proposes a stakeholder‐based, sustainable balanced scorecard conceptual framework coupled with a single‐measure organizational sustainability performance index to integrate the measures in a sustainable balanced scorecard. |
| Milne & Gray (2013) | 164 | J. Bus. Ethics | This study argues that the TBL and the Global Reporting Initiative are insufficient conditions for organizations to contribute to sustaining the Earth’s ecology. |
| Zhu et al. (2013) | 158 | J. Purch. Supply Manag. | This paper develops and empirically tests a theoretical model on the different types of institutional pressures motivating manufacturing enterprises to pursue green supply chain management practices and commensurate performance outcomes. |
| Cronin et al. (2011) | 151 | J. Acad. Mark. Sci. | As green marketing strategies become increasingly important to firms adhering to a TBL performance evaluation, this study seeks to better understand the role of ‘green’ as a marketing strategy. |
| Dao et al. (2011) | 125 | J. Strateg. Inf. Syst. | Using the resource-based view as its theoretical foundation, this manuscript develops an integrated sustainability framework, illustrating the integration of human, supply chain, and information technology resources to enable firms to develop sustainability capabilities. |
| Gleim et al. (2013) | 111 | J. Retail. | The research presented utilizes a critical incident qualitative study and two quantitative studies to examine the factors associated with nongreen purchase behaviors. |
| Schaltegger & Burritt (2010) | 111 | J. World Bus. | This paper reviews the literature on sustainability accounting from an information management perspective and distinguishes different interpretations of sustainability accounting. |
| Chabowski et al. (2011) | 107 | J. Acad. Mark. Sci. | Despite the progress made in the study of sustainability, there is a paucity of research on this topic in premiere marketing journals. To address this issue, the authors focus on marketing-related journals and assess the intellectual structure of sustainability research in detail. |
| Winter & Knemeyer (2013) | 99 | Int. J. Phys. Distrib. Logist. Manag. | This paper aims to provide a snapshot of the existing research and to suggest potential opportunities for academic inquiry related to the concept of sustainable supply chain management. |
| Devika et al. (2014) | 98 | Eur. J. Oper. Res. | This study develops a mixed integer programming model to bridge the gap by simultaneously considering the TBL of sustainability and the network design problem. |
| Hahn et al. (2015) | 92 | J. Bus. Ethics | This paper proposes a systematic framework for analyzing the tensions in corporate sustainability. |
| Gond et al. (2012) | 89 | Manage. Account. Res. | This paper addresses a gap and mobilizes a configuration approach to theorize the roles and uses of management control systems and sustainability control systems in the integration of sustainability within organizational strategy. |
| Foran et al. (2005) | 87 | Ecol. Econ. | In an analysis of the Australian economy, this study integrates financial input-output tables that describe the interdependencies between economic sectors with national social and environmental accounts to construct numerical TBL accounts for 135 discrete sectors. |
| Dixon & Clifford (2007) | 86 | J. Organ. Chang. Manage. | The purpose of this paper is to extend research on social and ecological entrepreneurship. It aims to examine how ecopreneurs can create an economically viable business while retaining their core environmental and social values. |
| Beske & Seuring (2014) | 81 | Supply Chain Manag. | The aim of this paper is to identify the key categories of sustainable supply chain management and the related practices that are required to meet the demands of sustainability and, therefore, to contribute to sustainability performance. |

A3. The 20 Most Highly Cited TBL Articles on the Environmental Aspect

| References | Citations | Journal | Summary |
| --- | --- | --- | --- |
| Pope et al. (2004) | 359 | Environ. Impact Assess. Rev. | This article seeks to provide some clarification by reflecting on the different approaches described in the literature as being forms of sustainability assessment and by evaluating them in terms of their potential contributions to sustainability. |
| Bocken et al. (2014) | 343 | J. Clean Prod. | Sustainable business model archetypes are introduced to describe groupings of mechanisms and solutions that may contribute to constructing a business model for sustainability. The aim of these archetypes is to develop a common language that can be used to accelerate the development of sustainable business models in research and practice. |
| Wu (2013) | 268 | Landsc. Ecol. | The main objectives of this paper are (1) to elucidate the key definitions and concepts of sustainability; (2) to examine the key definitions and concepts of landscape sustainability; and (3) to propose a framework for developing a science of landscape sustainability. |
| Govindan et al. (2013) | 223 | J. Clean Prod. | This paper explores sustainable supply chain initiatives and examines the problem of identifying an effective model based on the TBL approach (economic, environmental, and social aspects) for supplier selection operations in supply chains by presenting a fuzzy multicriteria approach. |
| Hubbard (2009) | 178 | Bus. Strateg. Environ. | This article proposes a stakeholder-based, sustainable balanced scorecard conceptual framework coupled with a single-measure organizational sustainability performance index to integrate the measures in a sustainable balanced scorecard. |
| Klewitz & Hansen (2014) | 156 | J. Clean Prod. | This paper analyzes the heterogeneous picture drawn by research over the past 20 years, with a focus on innovation practices including the different types of sustainability-oriented innovations and strategic sustainability behaviors of small and medium-sized enterprises, through an interdisciplinary systematic review over the 1987-2010 period. |
| Hahn & Kuhnen (2013) | 152 | J. Clean Prod. | This paper provides a review of 178 articles from 1999 to 2011 in journals related to business, management, and accounting. It finds that the current literature often still seems far from considering truly complete sustainability from the perspective of the TBL of sustainability. |
| Mori & Christodoulou (2012) | 147 | Environ. Impact Assess. Rev. | The purpose of this paper is to discuss the conceptual requirements for a city sustainability index and to review existing major sustainability indices/indicators in terms of the requirements. |
| Roca & Searcy (2012) | 122 | J. Clean Prod. | The purpose of this paper is to identify the indicators that are currently disclosed in corporate sustainability reports. |
| Foran et al. (2005) | 87 | Ecol. Econ. | In an analysis of the Australian economy, this study integrates financial input-output tables that describe the interdependencies between economic sectors with national social and environmental accounts to construct numerical TBL accounts for 135 discrete sectors. |
| Wiedmann et al. (2009) | 86 | J. Ind. Ecol. | This study incorporates all indicators related to environmental life cycle thinking, such as carbon, ecological, or water footprinting, into one common and consistent accounting and reporting scheme based on economic input-output analysis, and it develops a TBL accounting framework and software tool. |
| Hatt et al. (2006) | 85 | J. Environ. Manage. | This paper presents a review of Australian stormwater treatment and recycling practices and then considers that TBL assessment methodologies need to be trialed on stormwater recycling projects. |
| Nikolaou et al. (2013) | 75 | J. Clean Prod. | This paper proposes an integrated model for introducing corporate social responsibility and sustainability issues in reverse logistics systems as a means of developing a complete performance framework model. |
| Solomon et al. (2008) | 68 | Resour. Policy | This study provides a social map or landscape of industry in Australia. It identifies research policy and practice gaps and the emerging challenges faced by industry and society with the purpose of addressing the problem wherein the social dimension of the mining industry has been the least understood aspect of sustainable development in the TBL comprising the economy, environment and society. |
| Stylidis et al. (2014) | 66 | Tourism Manage. | Drawing on the TBL approach for tourism impacts (economic, sociocultural and environmental) and adopting a nonforced approach to measuring residents' perception of these impacts, this study explores the role of residents' place image in shaping their support for tourism development. |
| Lee et al. (2012) | 65 | J. Clean Prod. | To support the dynamic and multidimensional characteristics of product service systems (PSSs), this study employs system dynamics to cover the dynamics and the TBL to encompass the multidimensionality of PSS sustainability. The proposed approach is expected to effectively measure PSS sustainability through a comprehensive view. |
| Goerner et al. (2009) | 65 | Ecol. Econ. | This study combines thermodynamic, network, and information theoretic measures with research on real-life ecosystems to create a generalized, quantitative measure of sustainability for any complex matter/energy flow system. The current paper explores how this metric and its related concepts can be used to provide a new narrative for long-term economic health and sustainability. |
| Meehan & Bryde (2011) | 63 | Bus. Strateg. Environ. | The results of a survey of the sustainable procurement practices in 44 English-based UK housing associations, which are responsible for the provision of social housing, confirm prior research on other sectors that suggests 1) a failure to overcome inertia in relation to sustainable procurement and 2) that in the few examples where practices have been established, only the environmental element of the TBL is considered. |
| Schianetz et al. (2007) | 62 | Tourism Manage. | This study introduces the concept of learning tourism destinations and discusses the potential of system dynamics modeling as a tool for collective learning processes in the context of addressing the proliferation of learning organizations related to TBL sustainability. |
| Prajogo et al. (2012) | 49 | J. Clean Prod. | Based on institutional theory and the natural resource-based view, this study examines the relationship of two different organizational adoption motives (i.e., internal and external) with the perceived benefits of the TBL (i.e., environmental, social, and market) upon the adoption of ISO 14001. |

A4. The 20 Most Highly Cited TBL Articles on the Social Aspect

| References | Citations | Journal | Summary |
| --- | --- | --- | --- |
| Norman & MacDonald (2004) | 250 | Bus. Ethics Q. | This paper argues on both conceptual and practical grounds that the TBL is an unhelpful addition to current discussions of corporate social responsibility. |
| Milne & Gray (2013) | 164 | J. Bus. Ethics | This study argues that the TBL and the Global Reporting Initiative are insufficient conditions for organizations to contribute to the sustaining of the Earth’s ecology. |
| Hahn et al. (2015) | 92 | J. Bus. Ethics | This article proposes a systematic framework for analyzing the tensions in corporate sustainability. |
| Font & Harris (2004) | 78 | Ann. Touris. Res. | This paper explores the successes and challenges of five programs operating partly or wholly in developing countries that have introduced socioeconomic criteria to complete the TBL of sustainability. |
| Stylidis et al. (2014) | 66 | Tourism Manage. | This article proposes a stakeholder-based, sustainable balanced scorecard conceptual framework coupled with a single-measure organizational sustainability performance index to integrate the measures in a sustainable balanced scorecard. |
| Schianetz et al. (2007) | 62 | Tourism Manage. | This study introduces the concept of learning tourism destinations and discusses the potential of system dynamics modeling as a tool for collective learning processes in the context of addressing the proliferation of learning organizations related to TBL sustainability. |
| Spiller (2000) | 62 | J. Bus. Ethics | This paper adopts an international investment perspective and finds that it is possible for businesses and investors to achieve a TBL of environmental, social and financial performance. |
| Freeman & Hasnaoui (2011) | 57 | J. Bus. Ethics | The purpose of this paper is to discuss the conceptual requirements for a city sustainability index and to review existing major sustainability indices/indicators in terms of the requirements. |
| Kleine & von Hauff (2009) | 51 | J. Bus. Ethics | This article presents a description of a new management tool, a systematic method for implementing corporate social responsibility successfully based on a TBL approach to sustainability. It fills many of the existing gaps at the corporate level. |
| Gibson et al. (2012) | 50 | Sport Manag. Rev. | The purpose of this study is to examine six small-scale sports events and the work of a local sports commission in the context of the three pillars of sustainability: economic, social, and environmental. |
| Northcote & Macbeth (2006) | 48 | Ann. Touris. Res. | This paper develops a framework for employing the tourism yield concept in a multidimensional manner, particularly by applying it to various quality-of-life aspects promoted by sustainable development. |
| Menz (2010) | 44 | J. Bus. Ethics | This paper investigates the relationship between the valuation of Euro corporate bonds and the corporate social responsibility standards of mainly European companies. It finds that the risk premium for socially responsible firms was higher than that for nonsocially responsible firms, which goes against the traditional view that socially responsible firms are commercially successful and less risky. |
| Darcy et al. (2010) | 41 | J. Sustain. Tour. | This paper explores the concept of accessible tourism and its links with TBL sustainability. |
| Romijn & Caniels (2011) | 40 | Res. Policy | This paper traces how isolated Jatropha biofuel experiments have developed since early 2005 toward a sectoral production and innovation system and investigates the extent to which this system has been capable of developing and maintaining sustainable practices and producing sustainable outcomes. |
| Timur & Getz (2009) | 36 | Sustain. Dev. | This paper examines the concept of sustainable tourism development in urban destinations. Both qualitative and quantitative data from interviews conducted and questionnaires administered in Victoria and Calgary, Canada, and San Francisco, USA, are employed. Respondents representing the three clusters of the tourism industry, the local government and the host environment are examined on their interpretation of ‘sustainable tourism,’ sustainability goals and the barriers to achieving sustainable tourism in urban destinations. |
| Assaf et al. (2012) | 33 | Int. J. Hosp. Manag. | The results of this study, based on a sample of Slovenian hotels, support the hypotheses that more extensive reporting on environmental, social and financial issues leads to better hotel performance. |
| Jarvis et al. (2016) | 27 | Tourism Manage. | Using a novel approach, this case study of the Great Barrier Reef explores and quantifies the risks to visitor numbers utilizing tourist survey data supplemented by objective data from secondary sources. It identifies that economic, social and environmental factors affect trip satisfaction, which in turn is found to affect the likelihood of a tourist returning. |
| Pava (2007) | 27 | Bus. Ethics Q. | This study suggests that while limitations to TBL accounting exist, the critique of Norman and MacDonald is deeply flawed. |
| Mori & Yamashita (2015) | 27 | Habitat Int. | This paper presents a framework for a city sustainability index incorporating indicators in the environmental, economic and social dimensions. |
| Cornelissen et al. (2011) | 27 | Dev. South. Afr. | Drawing on a growing body of scholarship on legacy best and worst practices, this article discusses the economic, physical, infrastructural, social, political and environmental consequences of sport mega-events using insights from South Africa's hosting of the 2010 FIFA World Cup. |

A5. The 20 Most Highly Cited TBL Articles on the Operations Aspect

| References | Citations | Journal | Summary |
| --- | --- | --- | --- |
| Kleindorfer et al. (2005) | 579 | Prod. Oper. Manag. | This study reviews the ‘sustainability’ themes covered in the first 50 issues of *Production and Operations Management* and concludes with some thoughts on the future research challenges in sustainable operations management. |
| Wu & Pagell (2011) | 210 | J. Oper. Manag. | This study uses theory building through case studies to answer the following question: how do organizations balance short-term profitability and long-term environmental sustainability when making supply chain decisions under conditions of uncertainty? |
| Gimenez et al. (2012) | 174 | Int. J. Prod. Econ. | Different firms have implemented environmental programs (such as design to recycle, life cycle analysis or environmental certification) and social practices (such as programs aimed at improving employees' working conditions or projects to support the external community). This study aims to analyze the impact of these programs on each dimension of the TBL. |
| Klassen & Vereecke (2012a) | 127 | Int. J. Prod. Econ. | Drawing from related studies, this study focuses on better understanding key social management capabilities in the supply chain and linking these capabilities with responsibility, risk and performance. |
| Govindan et al. (2014) | 119 | Int. J. Prod. Econ. | This paper proposes a multiobjective optimization model by integrating sustainability in decision-making based on distribution in a perishable food supply chain network. It introduces a two-echelon location-routing problem with time windows for sustainable supply chain network design and for optimizing the economic and environmental objectives in a perishable food supply chain network. |
| Devika et al. (2014) | 98 | Eur. J. Oper. Res. | This study developed a mixed integer programming model to bridge the gap by simultaneously considering the TBL of sustainability in the network design problem. |
| Pagell & Gobeli (2009) | 88 | Prod. Oper. Manag. | This research provides a first examination of operational managers' experiences with and attitudes toward employee well-being and environmental issues, how these factors impact employee well-being and environmental performance, and how the three performance measures interrelate. |
| Hollos et al. (2012) | 87 | Int. J. Prod. Res. | This study tests the antecedents and implications of sustainable supplier cooperation according to the TBL. |
| Presley et al. (2007) | 77 | Int. J. Prod. Res. | The dual contribution of this paper includes investigating the design and development of a strategic sustainability evaluation framework and introducing the relationships of reverse logistics to the economic, environmental and social sustainability dimensions. |
| Gopalakrishnan et al. (2012) | 65 | Int. J. Prod. Econ. | This paper examines the drivers of sustainability and related key features based on the extant literature and a case study. Based on a case study of British Aerospace Systems, two resultant frameworks emerge that display the interdependence of the TBL and the essential elements required for a sustainable supply chain. |
| Dai & Blackhurst (2012) | 60 | Int. J. Prod. Res. | To help advance this area of research and to further integrate sustainability into the supplier selection modeling area, this study develops an integrated analytical approach, combining the analytical hierarchy process with quality function deployment, to enable the ‘voice’ of company stakeholders in the process. |
| Sarkis & Dhavale (2015) | 52 | Int. J. Prod. Econ. | In evaluating and selecting sustainable suppliers, this study takes a TBL (profits, people and the planet) approach and considers the business operations as well as the environmental impacts and social responsibilities of the suppliers. A novel methodological approach based on a Bayesian framework and Monte Carlo Markov chain simulation is developed to rank and select suppliers using specific selection objectives. |
| Wu et al. (2015) | 44 | Int. J. Prod. Econ. | The current study combines fuzzy set theory and the decision-making trial and evaluation laboratory method for a hybrid approach to investigating the effects of each criterion within green supply chain practices in the context of firms aggressively integrating green practices within their supply chain to balance TBL performance. |
| De Giovanni & Zaccour (2014) | 44 | Eur. J. Oper. Res. | This study considers a two-period closed-loop supply chain game where a remanufacturer appropriates returns' residual value. It finds that there is only a small region in which outsourcing the collection process contributes to the TBL. |
| Govindan et al. (2015) | 39 | Comput. Oper. Res. | There is still a gap in the quantitative modeling of sustainable supply chain network design that consists of the order allocation problem. This study fills this gap by simultaneously considering the sustainable order allocation problem in the sustainable supply chain network design as a strategic decision. A novel multiobjective hybrid approach called MOHEV is proposed. |
| Wilhelm et al. (2016) | 38 | J. Oper. Manag. | This study employs arguments from agency and institutional theory to explore the conditions under which first-tier suppliers will act as agents who fulfill the lead firm's sustainability requirements (i.e., the primary agency role) and implement these requirements in their suppliers' operations (i.e., the secondary agency role). |
| Taticchi et al. (2015) | 37 | Int. J. Prod. Res. | This paper reviews the existing literature related to decision-support tools and performance measurement for sustainable supply chain management. The article concludes that there is evidence that the research field is growing, and it calls for establishing the scope of current research. That is, the article identifies the need for integrated performance frameworks with new generation decision-support tools incorporating a TBL approach for managing sustainable supply chains. |
| Brandenburg & Rebs (2015) | 37 | Ann. Oper. Res. | This paper reviews 185 journal publications from the last 20 years that formalize issues related to sustainable supply chain management in quantitative models. It suggests that the interrelationships between the TBL dimensions are to be scrutinized in greater detail to avoid focused optimization of selected sustainability criteria. |
| Lai et al. (2013) | 33 | Int. J. Prod. Econ. | The literature remains unclear, particularly in the Chinese manufacturing context, as to how these reverse logistics practices are related to the organizational bottom line with respect to operational, financial, and social performance outcomes. Using survey data collected from Chinese export-oriented manufacturers, this study applies seemingly unrelated regressions to determine whether these six reverse logistics practices contribute to these three performance parameters simultaneously. |
| Ji et al. (2014) | 28 | Int. J. Prod. Econ. | Based on the TBL principles and double environmental medium regulations, this paper divides the environmental bottom line into the ecological impact and the carbon emissions bottom lines; the former is contingent on the ecological footprint, while the latter depends on the carbon footprint. |

A6. The 20 Most Highly Cited TBL Articles on the Technology Aspect

| References | Citations | Journal | Summary |
| --- | --- | --- | --- |
| Bocken et al. (2014) | 343 | J. Clean Prod. | Sustainable business model archetypes are introduced to describe groupings of mechanisms and solutions that may contribute to constructing a business model for sustainability. The aim of these archetypes is to develop a common language that can be used to accelerate the development of sustainable business models in research and practice. |
| Govindan et al. (2013) | 223 | J. Clean Prod. | This paper explores sustainable supply chain initiatives and examines the problem of identifying an effective model based on the TBL for supplier selection operations in supply chains by presenting a fuzzy multicriteria approach. |
| Klewitz & Hansen (2014) | 156 | J. Clean Prod. | This paper analyzes the heterogeneous picture drawn by research over the past 20 years, with a focus on innovation practices including the different types of sustainability-oriented innovations and strategic sustainability behaviors of small and medium-sized enterprises, through an interdisciplinary systematic review over the 1987-2010 period. |
| Hahn & Kuhnen (2013) | 152 | J. Clean Prod. | This paper provides a review of 178 articles from 1999 to 2011 in journals related to business, management, and accounting. It finds that the current literature often still seems far from considering truly complete sustainability from the perspective of the TBL of sustainability. |
| Dao et al. (2011) | 125 | J. Strateg. Inf. Syst. | Using the resource-based view as its theoretical foundation, this manuscript develops an integrated sustainability framework, illustrating the integration of human, supply chain, and IT resources to enable firms to develop sustainability capabilities. |
| Roca & Searcy (2012) | 122 | J. Clean Prod. | The purpose of this paper is to identify the indicators that are currently disclosed in corporate sustainability reports. |
| Chen et al. (2010) | 101 | Autom. Constr. | The resultant list of sustainable performance criteria provides team members with a new way to select a construction method, thereby facilitating the sustainable development of the built environment. |
| Wiedmann et al. (2009) | 86 | J. Ind. Ecol. | Indicators can be incorporated into one common and consistent accounting and reporting scheme based on economic input-output analysis, extended with data from all three dimensions of sustainability. This study introduces a TBL accounting framework and software tool and applies it in a case study of a small company in the United Kingdom. |
| Bordass et al. (2001) | 84 | Build. Res. Informat. | This study attempted to assess building performance by adopting the TBL. In addition, it argued that persistent chronic low-level problems need to be addressed if we are to move toward the TBL of more sustainable practice and create a basis of sound practice upon which innovations can flourish. |
| Nikolaou et al. (2013) | 75 | J. Clean Prod. | This paper proposes an integrated model for introducing corporate social responsibility and sustainability issues in reverse logistics systems as a means of developing a complete performance framework model. |
| Akadiri et al. (2013) | 69 | Autom. Constr. | Current building materials selection methods fail to provide adequate solutions to two major issues: assessment based on sustainability principles and the process of prioritizing and assigning weights to the relevant assessment criteria. This paper proposes a building materials selection model based on fuzzy extended analytical hierarchy process techniques with a view to providing solutions to these two issues. |
| Lee et al. (2012) | 65 | J. Clean Prod. | To support the dynamic and multidimensional characteristics of PSSs, this study employs system dynamics to cover the dynamics and the TBL to encompass the multidimensionality of PSS sustainability. The proposed approach is expected to effectively measure PSS sustainability from a comprehensive perspective. |
| Sayce et al. (2007) | 58 | Build. Res. Informat. | In developing its premise, this paper draws on the third of three surveys conducted by the authors spanning a decade and tracking investor attitudes toward ‘green’ and sustainable buildings within the context that the rise in concern for TBL sustainability is now embedded in many government and corporate policies but is still not integrated into UK investment practice. |
| Reza et al. (2011) | 56 | Constr. Build. Mater. | This paper utilizes TBL sustainability criteria for the selection of a sustainable flooring system in Tehran (Iran). The proposed approach provides a comprehensive evaluation system based on TBL criteria that are further divided into thirteen subcriteria. |
| Prajogo et al. (2012) | 49 | J. Clean Prod. | Based on institutional theory and the natural resource-based view, this study examines the relationship of two different organizational adoption motives (i.e., internal and external) with the perceived benefits of the TBL (i.e., environmental, social, and market) upon the adoption of ISO 14001. |
| Dubey et al. (2017) | 42 | J. Clean Prod. | This paper argues for the use of total interpretive structural modeling in sustainable supply chain management. |
| Darcy et al. (2010) | 41 | J. Sustain. Tour. | This paper explores the concept of accessible tourism and its links with TBL sustainability. |
| Allwood et al. (2008) | 41 | J. Clean Prod. | This paper presents a methodology for the scenario analysis of a large change to an entire sector. The approach includes the calculation of a “TBL graphic equalizer” to allow the exploration and evaluation of the tradeoff between economic, environmental and social impacts. |
| Sarkis et al. (2012) | 40 | J. Clean Prod. | This paper introduces a decision model and framework for subcontractor selection and team formation in the built environment based on the economic/business, social and environmental TBL aspects of sustainability. |
| Skouloudis et al. (2010) | 40 | J. Clean Prod. | This paper contributes to country-level analyses of nonfinancial reporting by assessing the quality and inclusiveness of TBL reports published by companies operating in Greece. To that end, a numerical scoring system is devised based on the de facto global standard on corporate nonfinancial reporting, the Global Reporting Initiative guidelines. |

A7. The 20 Most Highly Cited TBL Articles on the Engineering Aspect

| References | Citations | Journal | Summary |
| --- | --- | --- | --- |
| Kleindorfer et al. (2005) | 579 | Prod. Oper. Manag. | This paper reviews the "sustainability" themes covered in the first 50 issues of *Production and Operations Management* and concludes with some thoughts on future research challenges in sustainable operations management. |
| Bocken et al. (2014) | 343 | J. Clean Prod. | Sustainable business model archetypes are introduced to describe groupings of mechanisms and solutions that may contribute to constructing a business model for sustainability. The aim of these archetypes is to develop a common language that can be used to accelerate the development of sustainable business models in research and practice. |
| Govindan et al. (2013) | 223 | J. Clean Prod. | This paper explores sustainable supply chain initiatives and examines the problem of identifying an effective model based on the TBL for supplier selection operations in supply chains by employing a fuzzy multicriteria approach. |
| Gimenez et al. (2012) | 174 | Int. J. Prod. Econ. | Different firms have implemented environmental programs (such as design to recycle, life cycle analysis or environmental certification) and social practices (such as programs aimed at improving employees' working conditions or projects to support the external community). This study aims to analyze the impact of these programs on each dimension of the TBL. |
| Klewitz & Hansen (2014) | 156 | J. Clean Prod. | This paper analyzes the heterogeneous picture drawn by research over the past 20 years, with a focus on innovation practices including the different types of sustainability-oriented innovations and strategic sustainability behaviors of small and medium-sized enterprises, through an interdisciplinary systematic review covering the 1987-2010 period. |
| Hahn & Kuhnen (2013) | 152 | J. Clean Prod. | This paper provides a review of 178 articles from 1999 to 2011 in journals related to business, management, and accounting. It finds that the current literature often still seems far from considering truly complete sustainability from the perspective of the TBL of sustainability. |
| Klassen & Vereecke (2012b) | 127 | Int. J. Prod. Econ. | Drawing from related studies, basic constructs related to capabilities and risk are defined and used to underpin case research in five multinational firms. |
| Roca & Searcy (2012) | 122 | J. Clean Prod. | The purpose of this paper is to identify the indicators that are currently disclosed in corporate sustainability reports. |
| Govindan et al. (2014) | 119 | Int. J. Prod. Econ. | This paper proposes a multiobjective optimization model by integrating sustainability in decision-making based on distribution in a perishable food supply chain network. It introduces a two-echelon location-routing problem with time windows for sustainable supply chain network design and for optimizing the economic and environmental objectives in a perishable food supply chain network. |
| Chen et al. (2010) | 101 | Autom. Constr. | The resultant list of sustainable performance criteria provides team members with a new way to select a construction method, thereby facilitating the sustainable development of the built environment. |
| Azapagicm (2003) | 97 | Process Saf. Environ. Protect. | This paper proposes a general framework for a corporate sustainability management system that makes it possible to translate the general principles of sustainable development into corporate practice by providing systematic, step-by-step guidelines toward a more sustainable business. |
| Pagell & Gobeli (2009) | 88 | Prod. Oper. Manag. | This research provides a first examination of operational managers' experiences with and attitudes toward employee well-being and environmental issues, how these factors impact employee well-being and environmental performance, and how the three performance measures interrelate. |
| Hollos et al. (2012) | 87 | Int. J. Prod. Res. | This study tests the antecedents and implications of sustainable supplier cooperation according to the TBL. |
| Wiedmann et al. (2009) | 86 | J. Ind. Ecol. | Indicators can be incorporated into one common and consistent accounting and reporting scheme based on economic input-output analysis, extended with data from all three dimensions of sustainability. This study introduces such a TBL accounting framework and software tool and applies it in a case study of a small company in the United Kingdom. |
| Presley et al. (2007) | 77 | Int. J. Prod. Res. | The dual contribution of this paper includes investigating the design and development of a strategic sustainability evaluation framework and introducing the relationships of reverse logistics to the economic, environmental and social sustainability dimensions. |
| Nikolaou et al. (2013) | 75 | J. Clean Prod. | This paper proposes an integrated model for introducing corporate social responsibility and sustainability issues in reverse logistics systems by developing a complete performance framework model. |
| Akadiri et al. (2013) | 69 | Autom. Constr. | Current building materials selection methods fail to provide adequate solutions to two major issues: assessment based on sustainability principles and the process of prioritizing and assigning weights to the relevant assessment criteria. This paper proposes a building materials selection model based on fuzzy extended analytical hierarchy process techniques, with a view to providing solutions to these two issues. |
| Gopalakrishnan et al. (2012) | 65 | Int. J. Prod. Econ. | This paper examines the drivers of sustainability and related key features based on the extant literature and a case study. Based on a case study of British Aerospace Systems, two resultant frameworks emerge that display the interdependence of the TBL and the essential elements required for a sustainable supply chain. |
| Lee et al. (2012) | 65 | J. Clean Prod. | To support the dynamic and multidimensional characteristics of PSSs, this study employs system dynamics to cover the dynamics and the TBL to encompass the multidimensionality of PSS sustainability. The proposed approach is expected to effectively measure PSS sustainability from a comprehensive perspective. |
| Shahriar et al. (2012) | 61 | J. Loss Prev. Process Ind. | This study explores how interdependencies among various factors might influence analysis results, and it introduces fuzzy utility value to perform risk assessment for natural gas pipelines using TBL sustainability criteria. |
